# Reduced *Slc1a1* expression is associated with neuroinflammation and impaired sensorimotor gating and cognitive performance in mice: Implications for schizophrenia

**DOI:** 10.1371/journal.pone.0183854

**Published:** 2017-09-08

**Authors:** Parisa Afshari, Wei-Dong Yao, Frank A. Middleton

**Affiliations:** 1 Department of Neuroscience & Physiology, SUNY Upstate Medical University, Syracuse, NY United States of America; 2 Department of Psychiatry & Behavioral Sciences, SUNY Upstate Medical University, Syracuse, NY, United States of America; 3 Department of Biochemistry & Molecular Biology, SUNY Upstate Medical University, Syracuse, NY, United States of America; Niigata Daigaku, JAPAN

## Abstract

We previously reported a 84-Kb hemi-deletion copy number variant at the *SLC1A1* gene locus that reduces its expression and appeared causally linked to schizophrenia. In this report, we characterize the *in vivo* and *in vitro* consequences of reduced expression of *Slc1a1* in mice. Heterozygous (HET) *Slc1a1*^+/-^ mice, which more closely model the hemi-deletion we found in human subjects, were examined in a series of behavioral, anatomical and biochemical assays. Knockout (KO) mice were also included in the behavioral studies for comparative purposes. Both HET and KO mice exhibited evidence of increased anxiety-like behavior, impaired working memory, decreased exploratory activity and impaired sensorimotor gating, but no changes in overall locomotor activity. The magnitude of changes was approximately equivalent in the HET and KO mice suggesting a dominant effect of the haploinsufficiency. Behavioral changes in the HET mice were accompanied by reduced thickness of the dorsomedial prefrontal cortex. Whole transcriptome RNA-Seq analysis detected expression changes of genes and pathways involved in cytokine signaling and synaptic functions in both brain and blood. Moreover, the brains of *Slc1a1*^+/-^ mice displayed elevated levels of oxidized glutathione, a trend for increased oxidative DNA damage, and significantly increased levels of cytokines. This latter finding was further supported by *SLC1A1* knockdown and overexpression studies in differentiated human neuroblastoma cells, which led to decreased or increased cytokine expression, respectively. Taken together, our results suggest that partial loss of the *Slc1a1* gene in mice causes haploinsufficiency associated with behavioral, histological and biochemical changes that reflect an altered redox state and may promote the expression of behavioral features and inflammatory states consistent with those observed in schizophrenia.

## Introduction

Excitatory amino acid transporter 3 (EAAT3), known as excitatory amino acid carrier 1 (EAAC1) in rodents, is encoded by the *SLC1A1* gene and represents the major neuronal member of the excitatory amino acid transporter family encompassing EAATs 1–5 [1, 2]. EAAT3 is mostly expressed in hippocampus, followed by cerebral cortex, striatum and thalamus. More specifically, the highest transcript levels of EAAT3 in mice are seen in the pyramidal layer of hippocampal areas CA1 to CA4 and the granular layer of the dentate gyrus, where it is widely distributed in all neurons [2, 3, 4].

The EAAT3 protein has three main physiological functions: 1) uptake of synaptic glutamate and preventing the glutamate spillover from active synapses to extrasynaptic regions [1, 5, 6]; 2) providing glutamate as a precursor for gamma aminobutyric acid (GABA) synthesis [1, 7, 8]; and 3) neuronal uptake of cysteine, the rate-limiting substrate for synthesis of intracellular glutathione (GSH) in neurons. In fact, cysteine is transported by EAAT3 at a rate comparable to that of glutamate and with a roughly ten-fold greater affinity than that of the astrocyte transporters, EAAT1 and EAAT2 [9,10, 11,12].

GSH is the principal intracellular antioxidant in neurons, essential for the metabolism of reactive oxygen species (ROS). GSH depletion is associated with increased susceptibility to oxidative stress, which underlies the pathogenesis of neurodegenerative diseases and cognitive impairment [12, 13]. Given that mature neurons rely primarily on EAAT3 for uptake of cysteine and subsequent GSH synthesis [14, 15], it is plausible that this transporter performs essential neuroprotective roles in the brain. Consistent with this idea, EAAT3-null mice present with decreased neuronal GSH levels, increased indicators of neuronal oxidative stress, age-dependent neurodegeneration as well as cognitive impairment and behavioral abnormalities [11, 16, 17]. These mice are also more susceptible to neurodegeneration in models of ischemia, Parkinson’s disease, and aging [8, 11, 18, 19, 20, 21]. Interestingly, it has been observed that treatment of mice with N-acetylcysteine (NAC), a membrane-permeable cysteine precursor, attenuates or prevents the biochemical and behavioral abnormalities in EAAT3-null mice [11, 17, 19, 22].

A tightly regulated balance is known to exist between brain glutamatergic, redox, and inflammatory states, forming what has been termed a "central hub" where imbalances have been hypothesized to contribute to pathophysiological changes associated with schizophrenia and psychosis [23]. Besides confirming the neuroprotective role of EAAT3, some of the studies involving EAAT3-null mice report phenotypes consistent with an effect on GABAergic signaling, which has been strongly linked to some of the core features of schizophrenia [8]. Thus, the function of EAAT3 in glutamate transport, cellular responses to oxidative stress and inflammation, as well as GABA synthesis might form an attractive point of convergence for several pathophysiological models of schizophrenia.

The first group to generate an *Slc1a1*-knockout mouse strain, in which exon 1 of the gene was disrupted, reported that adolescent mice exhibited decreased spontaneous locomotor activity but did not report any neurodegeneration [24]. However, another group of researchers using descendants of the same knockout strain detected age-dependent behavioral abnormalities and brain atrophy in these mice. These changes were accompanied by reduced neuronal GSH levels and increased histochemical markers of neuronal oxidative stress in the hippocampus [11]. Similarly, Lee and colleagues detected significant deficits in context- and tone-related fear conditioning and Barnes maze performance in adolescent *Slc1a1*-knockout mice [16]. These reported effects on fear conditioning are highly consistent with parallel studies demonstrating increased translocation of EAAT3 from the cytosol to the plasma membrane in hippocampal CA1 neurons during fear conditioning [25]. Interestingly, many of the reported neurochemical, neuroanatomical and behavioral alterations could be reversed or prevented by administration of NAC to the knockout mice [11, 17, 19, 20], strongly suggesting that some of the abnormalities are related to the role that this protein plays in cysteine transport and the support of neuronal GSH metabolism, rather than glutamate transport *per se* [26, 27]. Additional evidence in support of this idea is found in studies of the *Gtrap3-18* gene, a negative modulator of EAAT3. Disruption of this gene in mice has been reported to increase the substrate affinity of EAAT3, which in turn elevates neuronal GSH levels and enhances neuronal resistance against oxidative stress [28, 29, 30]. Furthermore, *Gtrap3-18*-deficient mice have also shown improved performance in spatial/motor learning and memory tasks [31]. Overall, these findings highlight the importance of EAAT3 transporter in normal cognitive and neurochemical brain functions.

Schizophrenia is a disorder with pronounced alteration of cognitive and neurochemical processes, including glutamate, GABA and dopaminergic systems, as well as neuroanatomical changes. For example, reductions in whole brain, medial temporal and prefrontal lobe gray matter volumes have been described in subjects with schizophrenia and have been suggested to correlate with cognitive deficits in the subjects [32, 33, 34, 35]. These abnormalities have also been observed in at-risk individuals or on the first episode, implicating that they are not secondary to the disease or its treatment [36, 37, 38, 39]. There is also accumulating evidence that inflammation and immune dysfunction might contribute to the cognitive, negative, and positive symptoms in schizophrenia [40], with studies reporting abnormal levels of cytokines in both the brain and peripheral blood of patients with schizophrenia. In fact, these data have led to the development of a cytokine-based model of schizophrenia [41], which gains additional support from the characterization of the common genetic liability for schizophrenia associated with the major histocompatibility complex (MHC) and complement Component 4 (C4) genes according to large-scale genome-wide association studies (GWAS) [42].

We previously reported a novel 84-Kb hemi-deletion copy number variant (CNV) in *SLC1A1* that exhibits a large effect size causally linked to schizophrenia in a 5-generation family from the Pacific island of Palau [43]. This CNV segregated in an autosomal dominant manner and appeared to act as a loss of function allele, leading to reduced expression of functional *SLC1A1* and significantly impairing the ability of cells to transport glutamate and cysteine [44]. Since our report, other groups have also observed similarly localized *SLC1A1* CNVs in schizophrenia probands from other populations. Interestingly, these CNVs largely overlap the 5' end of the gene, suggesting that the expression level would be affected in those probands as well [45, 46, 47, 48, 49, 50]. When considered together, the combined genetic data suggest that uncommon CNVs of large effect involving *SLC1A1* may help explain a small proportion of the incidence of schizophrenia [44]. However, it is also possible that less severe genetic changes, such as minor allelic variation, could alter the expression of *SLC1A1* as well in subjects without CNVs.

While prior studies of *SLC1A1* knockout mice have yielded considerable insight into the function of EAAT3 protein, it is clear that the schizophrenic subjects with *SLC1A1* hemi-deletions are not well-modeled by complete gene deletions in mice. Therefore, to more closely mimic the effects of the *SLC1A1* hemi-deletion on an otherwise normal background, the current study examined the consequences of *SLC1A1* haploinsufficiency on brain and behavioral outcomes, using *Slc1a1*-heterozygous (HET) mice. Notably, the mice used in the present study were obtained from Jackson Laboratory and were generated by disruption of exon 7 of the gene. This construct allowed for inactivation of all potential isoforms of *Slc1a1* in mice [51], which prior studies did not accomplish. To our knowledge, there are no published studies on the *Slc1a1*-HET mice, and none that have attempted to examine behaviors using paradigms that are considered the most relevant for mouse models of schizophrenia, including prepulse inhibition (PPI) of the acoustic startle reflex, working memory or anxiety-like behavior [52]. Accordingly, the present study examined the above and other behavioral outcomes in *Slc1a1*-HET mice, with comparisons to KO mice for the behavioral studies included for comparative purposes.

Given the significance of oxidative stress in the pathophysiology of schizophrenia and the important role of EAAT3 in neuronal GSH synthesis, we tested whether *Slc1a1* haploinsufficiency could affect the redox state of glutathione (GSH/GSSG ratio) in the brain, as an indicator of cellular oxidative stress. Moreover, since excessive ROS can lead to DNA damage and ultimately cause cell death via apoptosis or necrosis [53], we also assessed the oxidative DNA damage in *Slc1a1*-HET mice. Oxidative stress can also lead to neuroinflammation and we hypothesized that *Slc1a1* haploinsufficiency would increase cytokine production in the brain. This hypothesis was tested using cytokine profiling of mouse brain tissue and human neural cell cultures that were exposed to oxidative stress or pro-inflammatory stimuli after knockdown or over-expression of *SLC1A1*. Finally, to examine the impact of *SLC1A1* haploinsufficiency on global brain function, we used RNA-Seq to determine whether this loss of function produces transcriptional effects on other schizophrenia-related, neuroimmune-related, and synaptic-related genes in the mouse brain tissue. Overall, our results indicate the presence of several behavioral, biochemical, and transcriptional changes in *Slc1a1*^+/-^ mice that are consistent with its potential utility in modeling schizophrenia and merit further study.

## Methods

### *Slc1a1*^*+/-*^ mice

All procedures were performed with approval of the Committee for Humane Use of Animals (CHUA) at SUNY Upstate Medical University and were in accordance with the guidelines for animal care established by the National Institute of Health. Male and female *Slc1a1*-HET mice were obtained from The Jackson Laboratory (Stock No. 024411, Bar Harbor, ME) and used as breeding pairs. These animals had been generated from crosses between *Slc1a1*-knockout (KO) mice on a C57BL/6NJ genetic background and wildtype (WT) mice of the same genetic background. In these knockout mice, exon 7 of the gene had been disrupted by Cre-loxP recombination. Specifically, the exon 7 flanked lox-P construct was introduced into C57BL/6N-derived JM8.N4 embryonic stem (ES) cells, and correctly targeted ES cells injected into B6(Cg)- Tyrc-2J/J (Stock No. 58) blastocysts. Chimeric male offspring were bred to C57BL/6NJ (Stock No. 005304) females and then to B6N.Cg-Tg(Sox2-cre)1Amc/J mice (Stock No. 014094) to remove the floxed neomycin and exon 7 sequences. The resulting mice were then bred to C57BL/6NJ mice to remove the Cre-expressing transgene and back-crossed an additional four generations to the C57BL/6N line and maintained as a stock line. Two adult male heterozygous mice (age 13 weeks) and four young adult (age 8 week) non-parous female heterozygous mice were ordered for breeding. Upon arriving at SUNY Upstate Medical University, each male was crossed with two of the females to yield several cohorts for the studies. The first cohort of offspring was not used for analysis. Mice were housed in a facility accredited by the Association for Assessment and Accreditation of Laboratory Animal Care (AAALAC). Rooms were temperature-controlled (22°C) and animals were maintained on a 12-h light/dark cycle with *ad lib* food and water. After crossing, litters were weaned on postnatal day (P) 22–23, ear punches obtained for DNA isolation and identification, and male and female offspring housed in separate cages (of 2–4 mice each). The behavioral, anatomical, and cytokine analyses were performed using the same groups of *Slc1a1*-HET, WT and KO mice produced during the on-site breeding process. Other biochemical and transcriptional profiling experiments utilized brain tissue from a different set of age-matched, experimentally-naïve WT and HET mice.

### Genotyping

Genotypes were determined by polymerase chain reaction (PCR) of ear DNA using the Jackson Laboratory recommended primer pairs. Each mouse was genotyped using two different PCR reactions with either the WT allele-specific primer pair (Forward: 5’- TACCCCAGTGACTCATCAGC-3’, Reverse: 5’- CATGGTGTTTACCAGCGTGA-3’), or the KO allele-specific primer pair (Forward: 5’-TTTCTACCTCGGGCCTAAGA-3’, Reverse: 5’-CGGTCGCTACCATTACCAGT-3’). Products were separated by 1.5% agarose gel electrophoresis and visualized by ethidium bromide staining. DNA from WT mice produced a single band corresponding to 269 bp, and DNA from KO mice produced a single band at 449 bp, while HET mice DNA produced both bands.

### Behavioral tests

A total of 34 mice were used in behavioral experiments (n = 9 HET males, 7 HET females, 5 WT males, 4 WT females, 4 KO males, 5 KO females). Testing was performed in a blind fashion. To minimize the chance of altering the behavioral responses by prior test history, the paradigms were performed in a specific order, which also helped reduce the effects of experimentally-induced anxiety or stress on later outcomes. The order of testing was as follows: Elevated Plus Maze (EPM), performed on P48-49; Y-Maze Spontaneous Alternation (P49-50); Spontaneous Activity in the Open Field (OF) test (P50-52); Novel Object Recognition (NOR) test, performed over two consecutive days (P51-52 and P52-53, respectively); and Pre-pulse Inhibition (PPI) of the Acoustic Startle Response (P53-54). Before each test, the animals were habituated to the behavioral testing room for at least one hour. To remove odor and residues, each apparatus was completely cleaned with 70% ethanol and dried between each test. Also, to reduce the effect of olfactory cues, male animals were always tested before the females.

#### Elevated plus maze (EPM) test

To assess anxiety-like behavior of the animals, the EPM test was conducted using the standard mouse apparatus from San Diego Instruments (San Diego, CA). This apparatus consists of two open and two closed arms, crossing perpendicular to each other at the center. To perform the test, each mouse was placed in the center zone with its head directed toward the open arm and allowed to explore freely. Following an initial 1-minute adaptation period, the number of entries and duration of time spent in each arm as well as the total distance traveled by the animal were recorded for 5 min. A video tracking system utilizing ANY-maze Software (Stoelting, Wood Dale, IL) was used for automated recording and scoring of the behavioral measures. The main contrasts of interests were the total duration of time spent in the open versus closed arms, as well as the total number of entries into the open versus closed arms.

#### Y-maze spontaneous alternation test

The prefrontal cortex has been suggested as the primary site of working memory and subjects with schizophrenia, who are considered to have prefrontal cortical dysfunction, have demonstrated deficits on a variety of working memory tests [54]. To assess spatial working memory, we utilized the Y-maze Spontaneous Alternation test. This test is based on the tendency of rodents to explore new environments. We used a custom-built Y-maze, with plain white fiberboard walls and flooring. The apparatus consisted of three walled arms, 15 inches long and 4.5 inches wide, and positioned at 120° angles from each other with a central triangular zone. Rodents placed in the Y-maze typically prefer to investigate a new arm of the maze rather than returning to one that was previously visited, unless they experience working memory dysfunction or exhibit perseverative behavior. Mice were placed in the center of the maze and allowed to freely explore the three arms for the duration of the test. After an initial 1-minute adaptation time, the total number of entries to the arms, the number of complete alternations (defined by consecutive entry into all three arms on consecutive choices irrespective of order), total distance traveled, and average speed of each animal were recorded for 5 minutes. ANY-maze software was used for recording and scoring entries and alternations. The fraction of alternation for each animal was calculated as: (number of alternations)/ (total number of arm entries– 2). As an additional factor, we also calculated the number of broken alternations, in which after visits to two consecutive arms, re-entry into one of them left the alternation incomplete.

#### Open field (OF) activity

This test was performed to assess the novel environment exploration, spontaneous locomotor activity and anxiety-related behavior (tendency to avoid the center zone) in the animals. The apparatus was from San Diego Instruments and consisted of a walled-chamber with four square non-transparent open field boxes. The animal was placed in the center of the apparatus under ambient light and allowed to move freely for 10 minutes, following an initial 1-minute adaptation period. Behavioral scoring was accomplished using video tracking with ANY-maze software. A preloaded geometric grid on the computer screen defined the areas of each box as the edge and center zones. The duration of time spent in the center zone, edge zone, total distance traveled and average speed were analyzed as our primary outcome measures. We also examined rotations, entries to the center zone, and the number and duration of freezing/immobile episodes.

#### Novel object recognition (NOR) test

To evaluate the object recognition memory of the animals, this test was performed over two consecutive days in the OF chamber, for habituation training and object recognition, respectively. One day after completion of the OF test, the mice were placed back into the chamber with the presence of two identical small objects (cubic metal nuts). Using ANY-maze software to divide the OF area accordingly, the number of entries and the duration of time spent in the object interaction zones or background (non-interacting) zones were recorded for 10 minutes, following an initial 1-minute adaptation time. On the second day, the mice were placed back into the same chamber and their behavior recorded for an additional 10 minutes. However, on this day, one of the previous objects was replaced with a novel object (a transparent acrylic cube with a similar size as the cubic metal nuts).

#### Prepulse inhibition (PPI) of the acoustic startle response

The PPI test is a cross-species measure of sensorimotor gating and its disruption is commonly reported to be an endophenotype of schizophrenia and psychosis [55, 56]. We assessed the potential effects of *Slc1a1* haploinsufficiency on PPI performance using commercial startle chambers and the accompanying Startle-Pro software (Med Associates, Fairfax, VT). Each chamber housed a clear non-restrictive container that rested on a platform motion sensor inside a dark, sound-insulated, and ventilated box. All acoustic startle reflex test sessions followed a 10-minute adaptation period during which the animal was left in the dark box without any acoustic stimuli, followed by a 5-minute acclimation period, during which background white noise (62 dB) was present but no other stimuli were presented and no data were collected.

Following this period, the data acquisition was performed over the course of 48 trials separated into three distinct blocks, with pseudorandom inter-trial intervals of 10-20s. Block I: consisted of 10 startle pulses only, where brief (38ms) bursts of sound (120dB) were presented at unpredictable temporal intervals. Block II: consisted of 28 trials of three different types presented in pseudorandom fashion. 4 trials were null, without any pre-pulse or startle stimuli; 4 trials were pre-pulse-only stimuli of 67–76 dB/18ms; 4 trials were startle-only stimuli of 120 dB/38ms, and the remaining 16 trials consisted of startle stimuli (120 dB/38ms), preceded by pre-pulses (67-76dB/18ms). As in Block I, all stimuli were delivered at unpredictable pseudorandom temporal intervals. Block III: consisted of the same stimuli presentations as Block I.

The PPI of startle reflex was calculated as the ratio of average peak values of startle reflexes when the startle stimuli were preceded by prepulses to the average peak values of startle reflexes when startle stimuli were presented alone, all during block II trials. This ratio was expressed as a percentage change in the ratio.

#### Statistical analysis

**Fig 1:**
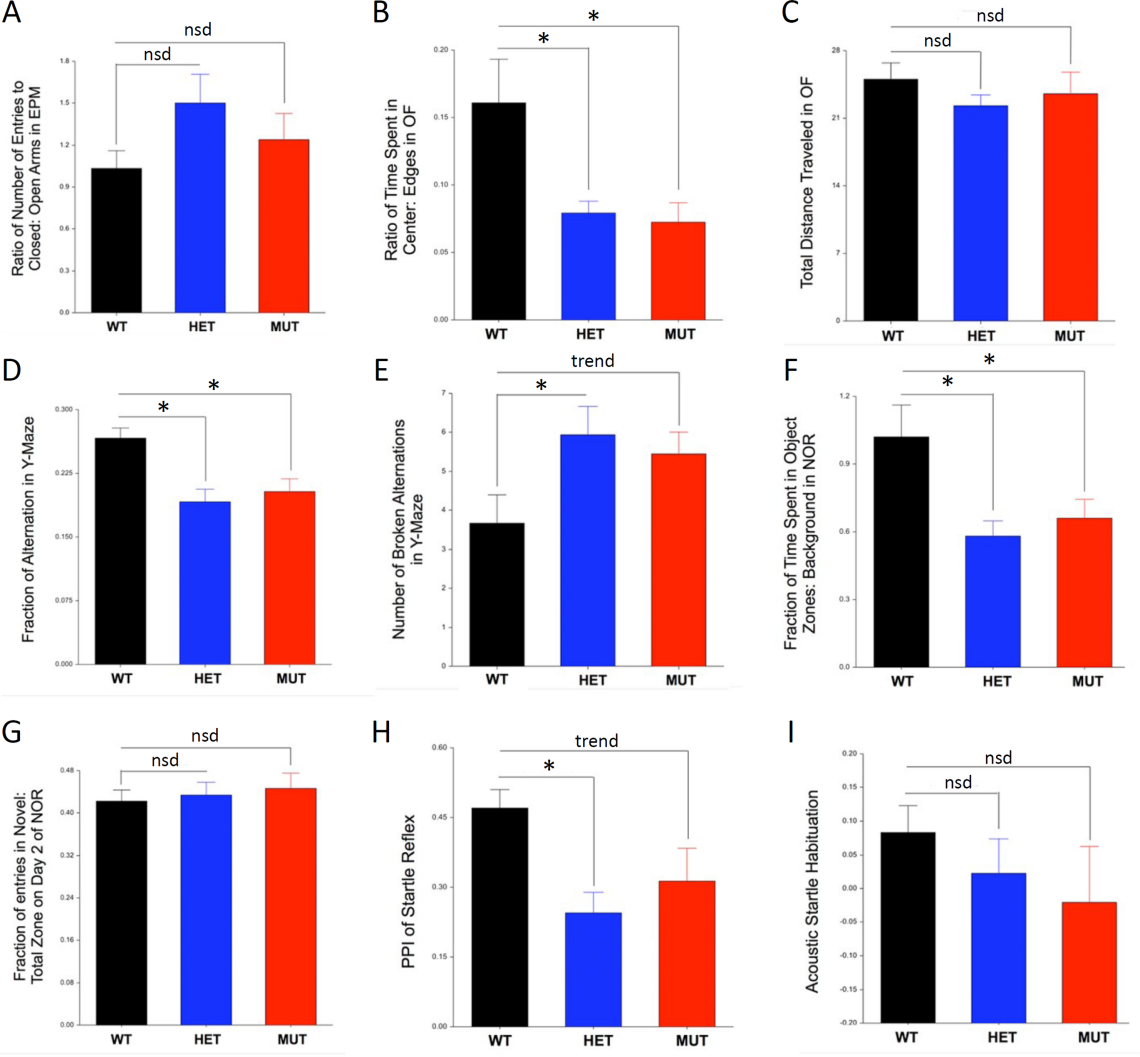
Behavioral tests showing significant differences due to *Slc1a1*-genotype. Behavioral data were analyzed for significance using a General Linear Mixed Model ANOVA incorporating two fixed factors (Genotype, Sex) and one random factor (litter Cohort) followed by a with a Dunnett’s post-test. There was no significant difference (nsd) in Elevated Plus Maze (EPM) performance, Total Distance Traveled in the Open Field (OF), Novel Object Recognition (NOR) performance, or Acoustic Startle Habituation (panels A, C, G, I), but 5 behaviors including Time Spent in the Center vs Edge of the OF, Y-Maze performance, Prepulse Inhibition (PPI), and overall object interaction time in the NOR (panels B, D, E, F, H) showed significant differences between the HET and WT mice (* = p < 0.05) and were also significantly changed or trending toward a similar change in comparisons of KO and WT mice. A trend was defined as a change in the same direction as the HET mice that exceeded the 95^th^ CL of the WT mean. Since there were no significant Sex x Genotype interaction effects and no main effect of Sex, histograms only indicate Genotype. Data are shown as mean ± SEM. *WT*, wildtype; *HET*, *Slc1a1*-heterozygous; *MUT*, *Slc1a1*-KO; *PPI*, prepulse inhibition.

Behavioral data were analyzed for significance with NCSS (v11.09; Kaysville, Utah, USA). A General Linear Mixed Model Analysis of Variance (GLMM ANOVA) was used with Genotype and Sex as fixed effects and litter Cohort as a random effect. We focused on detection of significant effects of Genotype, Sex, and Genotype x Sex interactions, with a Dunnett’s post-test used for planned comparisons of both HET and KO groups to the WT group. Results were visualized in tabular form **(Table 1)** as well as in histograms for the specific behavioral tests that showed a significant difference or a trend for difference between *Slc1a1*-HET, KO and WT mice **(Fig 1)**. We defined a trend as a change in the KO mice that was in the same direction as a significant change in the HET group that also fell outside the 95^th^ percentile confidence limit (CL) of the mean WT value. Since there were no significant Sex x Genotype interaction effects and no main effect of Sex, histograms for the behavioral studies only separate the data according to Genotype.

**Table 1:** Significant behavioral findings and trends in *Slc1a1*^*+/-*^ *and Slc1a1*^*-/-*^ *vs*. WT mice.

**Fraction of Alternation in Y-Maze**									
	**DF**	**Mean Square**	**F-Value**	**P-Value**	**Power**	**Dunnett's post-hoc test**			
**Sex**	1	4.26E-06	0	0.9674	0.0502	**Comparison**	**Count**	**Mean**	**Signif?**
**Genotype**	2	0.0167	6.67	0.0048	0.8774	**WT**	9	0.2674	
**Sex** * **Genotype**	2	5.06E-05	0.02	0.9800	0.0527	**HET**	16	0.1890	Yes
**Cohort**	3	0.0058	2.33	0.0989		**MUT**	9	0.2098	Yes
**Number of Broken Alternations in Y-Maze**									
	**DF**	**Mean Square**	**F-Value**	**P-Value**	**Power**	**Dunnett's post-hoc test**			
**Sex**	1	0.1103	0.03	0.6102	0.0787	**Comparison**	**Count**	**Mean**	**Signif?**
**Genotype**	2	15.7397	3.58	0.0017	0.9401	**WT**	9	3.5153	
**Sex** * **Genotype**	2	7.5529	0.06	0.9422	0.0580	**HET**	16	5.9399	Yes
**Cohort**	3	17.4752	1.57	0.2204		**MUT**	9	5.1586	Trend*
**Ratio of Time Spent in Center: Edges in OF**									
	**DF**	**Mean Square**	**F-Value**	**P-Value**	**Power**	**Dunnett's post-hoc test**			
**Sex**	1	0.0009	0.027	0.9674	0.0502	**Comparison**	**Count**	**Mean**	**Signif?**
**Genotype**	2	0.0281	8.34	0.0048	0.8774	**WT**	9	0.1696	
**Sex** * **Genotype**	2	0.0002	1.72	0.9800	0.0527	**HET**	16	0.0773	Yes
**Cohort**	3	0.0053	3.97	0.0989		**MUT**	9	0.0725	Yes
**Fraction of Time Spent in Object Zones: Background in NOR**									
	**DF**	**Mean Square**	**F-Value**	**P-Value**	**Power**	**Dunnett's post-hoc test**			
**Sex**	1	0.1123	1.06	0.9674	0.1682	**Comparison**	**Count**	**Mean**	**Signif?**
**Genotype**	2	0.6176	5.85	0.0048	0.8295	**WT**	9	1.0412	
**Sex** * **Genotype**	2	0.0101	0.1	0.9800	0.0630	**HET**	16	0.5676	Yes
**Cohort**	3	0.0385	0.36	0.0989		**MUT**	9	0.6758	Yes
**PPI of Startle Reflex**									
	**DF**	**Mean Square**	**F-Value**	**P-Value**	**Power**	**Dunnett's post-hoc test**			
**Sex**	1	0.0319	0.9	0.3508	0.1499	**Comparison**	**Count**	**Mean**	**Signif?**
**Genotype**	2	0.1343	3.81	0.0360	0.6380	**WT**	9	0.4754	
**Sex** * **Genotype**	2	0.0020	0.06	0.9462	0.0575	**HET**	16	0.2546	Yes
**Cohort**	3	0.0015	0.04	0.9881		**MUT**	9	0.3052	Trend*

* Trend defined as a change in the same direction as HET that exceeded the 95^th^ CL of the WT mean

### Brain morphology measurements

#### Tissue preparation and histology

The brain tissues from selected mice tested in behavioral phenotyping were used for morphometric measurements (n = 4 HET males, 4 HET females, 2 WT males, 3 WT females). At age P55-75, animals were sacrificed by CO_2_ asphyxiation and weighed before decapitation. Brains were then rapidly removed, weighed, snap-frozen in dry ice and stored at −80°C. We first compared the fresh frozen brain wet weight (g) and calculated brain to body weight ratios in HET and WT mice. These data were analyzed using a GLMM ANOVA incorporating Genotype and Gender as fixed effects and litter Cohort as a random effect followed by a Scheffé post-hoc test. To facilitate regional comparisons, the frozen left hemispheres were sectioned in the sagittal plane (30 μm) on a cryostat (Leica Microsystems, Buffalo Grove, IL) and mounted on SuperFrost Plus slides, with each slide containing up to 5 sections. Slides were stored at −80°C until used. For Nissl staining, sections were rapidly thawed, fixed in 4% paraformaldehyde in PBS, stained in 0.5% cresyl violet (wt/vol), dehydrated by submersion in graded ethanols (70, 90 and 100% x 2; vol/vol), placed in xylene and coverslipped using VectaMount mounting medium (Vector Laboratories, Burlingame, CA).

#### Morphometric measurements

Two sagittal brain sections were selected by two independent raters from all available sections to contain matching levels of the rostral dorsomedial and ventromedial prefrontal cortex (DMPFC and VMPFC) regions, and four sections were chosen from all available sections to contain the matching regions of the dorsal hippocampus. Representative images were then taken from each of the chosen sections blind to mouse genotype using a 4X objective on a Leica AS LMD microscope. NIH ImageJ software was used for measurements in a blind fashion. To test for significant differences in the regional morphometric measures while controlling for repeated sampling of the same mouse and a more robust control of the Type I error rate, we used a two-way (Sex x Genotype) repeated measures ANOVA combining all available measurements for each of the layers as well as the total cortical thickness, followed by a Scheffé post-hoc test, with alpha = 0.05.

#### Prefrontal cortex

The total cortical thickness of layers II–VI, as well as the widths of individual cortical layers II/III, IV, V, and VI were measured by a blinded rater in duplicate on two different sections for each animal, in both the DMPFC and VMPFC areas.

#### Hippocampus

Multiple measurements of hippocampal regional morphometry were also performed in a blind fashion, and included the total area of the hippocampal formation, the subareas of the hippocampus proper and dentate gyrus (DG), and the ratio of the hippocampus proper area to the DG area. We also measured the length of the stratum pyramidale (pyramidal cell layer) of the entire cornu ammonis (CA), the length of the stratum granulosum (granule cell layer) in the DG, the total length of these combined, and the ratio of CA to DG lengths. These measurements were obtained on 4 matching section levels through the hippocampus of each animal. Similar to the frontal cortex analysis, to test for significant differences in area or length, we used a two-way (Sex x Genotype) repeated measures ANOVA combining all available measurements.

### RNA sequencing

Whole transcriptome profiling was performed on blood and brain tissues of 5 male HET and 5 male WT mice (P48-52), using a stranded RNA-Sequencing (RNA-Seq) approach in the SUNY Molecular Analysis Core (SUNYMAC) facility at Upstate Medical University. Both the blood and brain tissues were supplied by Jackson Laboratories and arrived on dry ice and were immediately stored at -80°C until use. For the blood, RNA was isolated using Qiazol reagent followed by clean up with the RNeasy Mini Kit (Qiagen, Germantown, MD). For brain tissue RNA purification, the brains were ground into a fine powder in liquid nitrogen using a mortar and pestle, which was placed on dry ice. The powder was split into three equal aliquots for molecular studies. For RNA sequencing, the total RNA from one aliquot was purified using RNeasy Kit (Qiagen). All RNA samples were subjected to quantification and integrity assessment using the Agilent RNA 6000 Nano Kit (Agilent Technologies, Santa Clara, CA). All of the samples had RIN numbers of at least 9.8 (average 9.98). Libraries were generated using 1.5 ug RNA from each sample according to the TruSeq Stranded Total RNA with Ribo-Zero Globin Kit (Illumina, San Diego, CA). The samples were sequenced using an Illumina NextSeq 500 instrument with 1 x 75 bp single end reads, and a targeted average depth of coverage > 20 million reads per sample. After obtaining the raw sequence reads, FASTQ files were uploaded into BaseSpace (Illumina; https://basespace.illumina.com/apps) for quality control, alignment and quantification of reads to the mm10 build of the mouse genome using the STAR aligner, and differential expression analysis using DE-Seq2 analysis tools within the RNA Express v1.0 application. Raw FASTQ RNA-Seq files for this study are available in the NCBI Gene Expression Omnibus (Accession number: GSE103194). Differences in expression between the HET and WT mice were considered significant after adjusting for multiple testing based on a q value < 0.05. To determine the functional relevance of the most robustly changed genes, we first filtered the genes based on q < 0.01 and an absolute difference exceeding +/- 25% (i.e., log2 change > +/- 0.322) for the brain, or +/- 2-fold for the blood (i.e., log2 change > +/- 1.0) and uploaded the gene lists to the STRING Consortium Database (version 10; http://string-db.org) for Gene Ontology (GO) and Kyoto Encyclopedia of Genes and Genomes (KEGG) functional pathway enrichment analysis and Ingenuity Pathway Analysis (IPA) software (Qiagen) for upstream regulator analysis. Upstream regulator analysis identifies whether common transcriptional modifiers could explain the observed effects on multiple sets of genes. Predictions from the known relationship of the modifier to the outcome are made and examined to determine their overall consistency with the observed effects. In this report, we selected the 20 most robustly predicted upstream regulators for each tissue.

### Measurement of glutathione levels

A second aliquot of finely ground powder from the whole brain tissues used for RNA sequencing was used for measurement of glutathione in a total of 10 male mice (n = 5 HET, 5 WT, P48-52, naïve to behavioral testing). The levels of total and oxidized (GSSG) glutathione were measured using the colorimetric Glutathione (GSSG/GSH) Detection Kit (ADI-900-160, Enzo Life Sciences, Farmingdale, NY), according to manufacturer instructions. Briefly, the ground brain tissues were homogenized in ice-cold 5% (wt/vol) metaphosphoric acid (20 mL/g tissue). To measure the oxidized glutathione levels, the samples were treated with 2M 2-vinylpyridine (2 uL per 100 uL of sample homogenate) and incubated at room temperature for 60 minutes prior to being processed. The total and oxidized glutathione samples were diluted 40x and 5x, respectively and read in duplicates. The absorbance was read on a Synergy 2 plate reader (BioTek, Winooski, VT) at 405 nm every minute for a total duration of 11 minutes. The concentrations were calculated based on the slope of absorbance change over the 11 reads obtained at 1-minute intervals after interpolation of the absorbance slope curve of the standard samples provided with the kit. The reduced glutathione (GSH) concentration was calculated by subtracting oxidized glutathione from the total glutathione concentration. Statistical comparisons were made between the reduced to oxidized glutathione ratios (GSH/GSSG) in *Slc1a1*-HET and WT samples using an unpaired Student's t-test.

### Detection of oxidative DNA damage marker

To determine whether *Slc1a1* haploinsufficiency induces oxidative DNA damage, the level of 8-hydroxy-2′-deoxyguanosine (8-OHdG), a sensitive index of such damage in the brain, was measured. For this purpose, DNA samples from the same homogenized brain tissues mentioned earlier (from 5 HET male and 5 WT male mice) were extracted using the DNeasy Blood & Tissue Kit (Qiagen). Subsequently, 300 ng of each DNA sample was tested in the colorimetric EpiQuik 8-OHdG DNA Damage Quantification Direct Kit (P-6003-48, Epigentek, Farmingdale, NY), following manufacturer instructions. The absorbance was measured on a Synergy 2 plate reader (BioTek) at 450 nm. The results were expressed as absolute quantification (pg) according to the recommended formula and using the standard samples provided with the kit. The calculated 8-OHdG levels were averaged in each genotype group and comparisons were made using an unpaired Student's t-test.

### Apoptosis screening

We used a commercial Luminex-based magnetic bead assay (MILLIPLEX MAP Active Caspase 3 and MILLIPLEX MAP Phospho BAD, Millipore, Billerica, MA) to determine the levels of active caspase-3 and phospho-BAD in the brains of a subset of the same *Slc1a1*-HET and WT mice used for behavioral phenotyping and morphometry (n = 4 males and 4 females per genotype, n = 16 total). For these assays, samples of the DMPFC and frontal cortex were dissected at -20°C with a 22G needle and homogenized in 300 uL of ice cold Lysis Buffer from the MILLIPLEX MAP Cell Signaling Buffer and Detection Kit (Millipore) with 1% protease inhibitor added. These lysates were diluted 1:1 with 300 uL of Assay Buffer 2 from the same kit, and ultrafiltered with a sterile low protein-binding 0.2 micron syringe filter (Pall, Port Washington, NY). 25 uL of each filtered lysate was used for quantification of caspase-3 and phospho-BAD per manufacturer instructions, using highly specific antibody-coupled magnetic beads (Caspase 3: 46-604MAG, Phospho-BAD: 46-694MAG, Millipore). To control for total protein amounts, we included a third antibody-coupled magnetic bead to quantify beta-tubulin (46-713MAG, Millipore). After the primary antibody incubations, samples were washed, incubated with detection antibody, washed again, and then each well was filled with 150 uL Assay Buffer. Plates were read with a Bio-Plex 200 instrument (Bio-Rad, Hercules, CA). The median fluorescent intensity (MFI) was measured for at least 100 beads per target protein in each well, and the background intensity was subtracted using a control well. Ratios of the active caspase-3 to beta-tubulin and phospho-BAD to beta-tubulin were then compared between the mice in different groups. Comparisons between the groups were performed using a 2-way ANOVA (Sex x Genotype), with a Scheffé post-hoc test used to control for possible non-normality of the ratio data.

### Cytokine profiling

We also sought to examine the possible evidence for an increased inflammatory state in the brains of the *Slc1a1*-HET mice. This was performed using the same brain lysate samples prepared for the apoptosis assays. For the profiling, we used the MILLIPLEX MAP Cytokine/Chemokine Magnetic Bead Panel (RECYMAG65K27PMX, Millipore) to simultaneously screen for changes in 27 cytokines using highly specific antibody-coupled magnetic beads. The cytokines included in this assay were EGF, Eotaxin/CCL11, Fractalkine, G-CSF, GM-CSF, GRO/KC, IFNgamma, IL-1alpha, IL-1beta, IL-2, IL-4, IL-5, IL-6, IL-10, IL-12 (p70), IL-13, IL-17A, IL-18, IP-10, Leptin, LIX, MCP-1, MIP-1alpha, MIP-2, TNF-alpha, RANTES and VEGF. Plates were prepared per manufacturer instructions using 25 uL of each lysate per well. Briefly, each well of the 96-well assay plate was blocked with wash buffer for 10 minutes before use. The assay standards included with the kit were prepared by 4-fold serial dilutions. After adding the experimental or standard samples to the appropriate wells and serum matrix to the background wells, the antibody-immobilized beads were dispensed into each well and incubated with agitation overnight at 4°C. The next day, the wells were washed and incubated with a detection antibody, followed by streptavidin, additional washing, and resuspension in sheath fluid for reading on the Bio-Plex 200 plate reader. To calculate the cytokine concentrations (pg/mL) in the samples and controls, the MFI was analyzed using a 5-parameter logistic method after background correction. To control for different amounts of total protein in the lysates, the cytokine values were normalized to the levels of β-tubulin previously determined for each lysate. Comparisons between the groups were performed using a 2-way ANOVA (Sex x Genotype), with a Scheffé post-hoc test to control for possible non-normality.

### Effects of knockdown or over-expression of *SLC1A1* on neural cytokine production

Based on the results from the cytokine profiling in *Slc1a1*-HET and WT mice, we next evaluated how the expression level of *SLC1A1* transcript might affect the response of differentiated human neuroblastoma SK-N-SH cells to oxidative stress or pro-inflammatory stimuli.

#### Cell culture

Aliquots of the human SK-N-SH cell line were obtained from Dr. Yanli Zhang-James (SUNY Upstate). Cells were cultured in D-MEM medium (Thermo Fisher Scientific, Waltham, MA), supplemented with 10% fetal bovine serum (FBS) and 100 U/mL Penicillin-Streptomycin (Thermo Fisher Scientific) and incubated in humidified atmosphere at 37°C with 5% CO_2_. For differentiation, cells were plated into 0.1% gelatin-coated dishes at a density of 10 x 10^3^ cells per cm^2^ and grown as a monolayer in DMEM+. One day after plating, 10 μM retinoic acid (Sigma-Aldrich, St. Louis, MO) dissolved in dimethyl sulfoxide (DMSO) was added to the culture medium. The medium was replaced on alternate days and the cells were allowed to differentiate for 2 weeks to adopt a neural-like phenotype.

#### Transfection of *SLC1A1* shRNA or cDNA

All transfections were performed using the Nucleofection platform with the 4-D Nucleofector System (Lonza, Walkersville, MD) and Cell Line Nucleofector Kit V (VCA-1003, Lonza). For the knockdown studies, a cocktail of three validated human MISSION *SLC1A1* shRNAs (SHCLNG-NM_004170, Sigma-Aldrich) or pLKO1-puro non-target shRNA (Sigma-Aldrich) as a negative control were used. For the overexpression studies, GFP-tagged full-length *SLC1A1* construct was used. The construct contained the sequence-verified WT human *SLC1A1* ORF (1575 bp) subcloned into pCMV6-AC-GFP vector (PS100040, Origene, Rockville, MD). According to manufacturer instructions, PmaxGFP plasmid was used as the positive control for measuring the nucleofection efficiency. On the day of transfection, differentiated SK-N-SH cells were harvested by trypsinization and counted. For each transfection condition, 1 x 10^5^ cells were centrifuged in an individual Eppendorf tube at 90g for 10 min and the Nucleofector Solution was added. After adding either 0.2 ug of cDNA construct or 20 nM of *SLC1A1*- or non-target shRNA to each tube, the nucleofection procedure was performed and nucleofected cells were plated in a 96-well plate using eight replicate wells for each transfection condition (i.e., a total of 32 wells). Cells were maintained for 48 hours before checking the fluorescence intensity with a microscope to estimate transfection efficiency. For these studies, the transfection efficiency was estimated at 80% (data not shown).

#### Quantitative confirmation of overexpression and knockdown of *SLC1A1*

48 hours after nucleofection, the RNA from two wells in each transfection condition was isolated using the RNeasy Mini Kit (Qiagen) and 500 ng of RNA was used to synthesize cDNA using the Quantitect Reverse Transcription Kit (Qiagen). To quantitatively confirm the *SLC1A1* overexpression or knockdown in nucleofected human SK-N-SH cells, real-time RT-qPCR was performed in triplicate reactions with a custom-designed primer pair that amplified a 102-bp sequence in exon 10 of the gene (left primer: 5’-ATTCGTGTTACCCGTTGGTG-3’; right primer: 5’-CCCAAGTCCAGGTCATTCAA-3’). Thermal cycling was performed using 5 uL of cDNA from each sample, with 0.5 uL of each forward and reverse primers in a standard qPCR reaction in 20 uL volumes using LightCycler 480 SybrGreen I Master Mix (Roche, Indianapolis, IN) and the following conditions: 95°C for 5 min, then 40 cycles of 95°C for 15 sec, 59°C for 10 sec and 72°C for 15 sec. Quantification was performed using the delta delta Ct method, with human *RPLP1* used as the reference gene. Resulting data were log2 scaled and comparisons were made between the control, cDNA, and shRNA samples using a Welch's t-test.

#### Treatment of cells with oxidative stress or proinflammatory stimuli

In each set of transfected cultures, SK-N-SH cells were treated with either: 1) lipopolysaccharide (LPS, Sigma-Aldrich) at a concentration of 100 ng/mL, or 2) rotenone (Sigma-Aldrich) dissolved in 0.01% DMSO at a concentration of 100 nM, or 3) 0.01% DMSO as the vehicle. Each experimental condition was performed in duplicate wells and maintained for 48 hours.

#### Cytokine profiling

The supernatant in each well was collected and centrifuged. Using the Human High Sensitivity T-Cell Magnetic Bead Panel (HSTCMAG28SPMX21, Millipore), the levels of 21 different cytokines were quantified in each supernatant. The cytokines included in this assay were Fractalkine, GM-CSF, IFNgamma, IL-1beta, IL-2, IL-4, IL-5, IL-6, IL-7, IL-8, IL-10, IL-12 (p70), IL-13, IL-17A, IL-21, IL-23, ITAC, MIP-1alpha, MIP-1beta, MIP-3alpha and TNFalpha. Plates were prepared and analyzed as described earlier. The MFI values were adjusted for the background and the replicates averaged across the two plasmid control conditions (scrambled shRNA and PmaxGFP) for display purposes. Statistical comparisons were made using a 2-way ANOVA incorporating 3 different treatments (DMSO, rotenone or LPS) and 3 different plasmids (control, cDNA, shRNA). Hierarchical clustering was then performed to help visualize changes in cytokine levels across the conditions, and histogram plots made for selected proteins.

## Results

### Behavioral outcomes

#### Reduced *Slc1a1* increases anxiety-like behavior

Since anxiety is a highly prevalent comorbidity of schizophrenia [55], we investigated whether *Slc1a1*-HET mice would exhibit more anxiety-like behavior, compared to WT mice. Our results with the Elevated Plus Maze (EPM) showed a non-significant increase in time spent in the closed versus open arms of the maze, but this difference did not achieve significance **(Fig 1; Table 1)**. However, we did observe significantly reduced Center: Edge time in the Open Field (OF) task in both HET and KO (MUT) mice along with no difference in Total Distance Traveled in the OF. In addition, we found no significant effects of Sex, or Sex x Genotype interactions, in either of these measures. Notably, the magnitude of the effect was equivalent in the KO and KO mice. Together, these observations are consistent with a likely increase in anxiety-like behavior as a result of reduced *Slc1a1* expression.

#### Reduced *Slc1a1* impairs working memory

Impairment of working memory, including its spatial domain, is considered one of the core cognitive impairments in schizophrenia [56]. Working memory is often used to monitor prior choices in order to guide future actions. We attempted to assess the working memory of *Slc1a1*-HET and KO mice using the Y-maze spontaneous alternation test. *Slc1a1*-HET and KO mice showed significantly and equivalently reduced fractions of alternations compared to WT mice **(Fig 1; Table 1)** along with equivalently increased broken alternations compared to WT mice **(Fig 1; Table 1)** and no significant effects of Sex or Sex x Genotype interactions. Our results suggest that working memory is disrupted in mice with reduced expression of the *Slc1a1* gene.

#### Reduced *Slc1a1* does not influence locomotor activity of mice

Locomotor hyperactivity is sometimes reported in mouse models of schizophrenia and psychosis [57, 58]. Thus, we examined the locomotor activity of *Slc1a1*-HET and WT mice as measured by the total distance traveled in the OF test and the other tests. As already noted, our data revealed no significant effect of Genotype or Sex on the total distance traveled in the OF test **(Fig 1)** or in the EPM, NOR, and Y-Maze tests (not shown). The lack of any effect on overall locomotor activity is consistent with previous data on the *Slc1a1* KO mice.

#### Reduced *Slc1a1* decreased exploration of objects

In the Novel Object Recognition (NOR) test, the ratio of exploration time for the novel object *vs*. familiar object was not significantly different in *Slc1a1*-HET and WT mice, and there was no preference for the novel object over the familiar object in either of the genotypes (**Fig 1**). This lack of preference could not be attributed to the position of the objects inside the boxes, as the ratio of time spent in the object zones was similar in habituation and recognition test days in both genotypes (data not shown). However, we did observe a significant main effect of Genotype on exploratory activity toward the object zones in general (**Fig 1, Table 1**), as measured by total duration of time spent in either object zone compared to background, which was significantly reduced in HET mice and showed the same trend in KO mice. For simplicity, the exploratory activity data from habituation and test days were combined and displayed in a single histogram. These observations suggest that there is a reduction of general interest or attention for the objects in *Slc1a1*-HET mice, compared to the WT mice.

#### Reduced *Slc1a1* impairs prepulse inhibition

As a measure of sensorimotor gating, the disruption of prepulse inhibition (PPI) of the acoustic startle reflex is commonly accepted as an endophenotype of schizophrenia [57, 59]. We detected a significant effect of Genotype on PPI (F_(2,33)_ = 3.81, p = 0.036) with a trend for decreased PPI in both HET and KO mice (post-hoc p = 0.0243 and 0.1548 respectively) **(Fig 1; Table 1)**. No significant effects of Sex or Sex x Genotype interaction were observed. These results suggest that *Slc1a1*-HET mice exhibit a deficit in sensorimotor gating function, a fundamental form of information processing that appears to be deficient in different models of schizophrenia [60].

### Morphological changes

#### No overall difference in brain mass due to genotype

Despite a tendency for females to have lower brain weights than males, there was no significant difference in overall fresh frozen brain weight due to Genotype (F_1,11_ = 0.39, p = 0.549), Sex (F_1,11_ = 2.88, p = 0.128), or the interaction of Genotype x Sex (F_1,11_ = 0.01, p = 0.931) (data not shown). However, Brain:Body Weight ratios did show a significant effect of Sex (F_1,11_ = 12.04, p = 0.008), but no effect of Genotype (F_1,11_ = 0.41, p = 0.541) or Genotype x Sex interaction (F_1,11_ = 1.25, p = 0.296) (not shown).

#### The thickness of DMPFC is reduced in *Slc1a1*^+/-^ mice but VMPFC thickness and hippocampal size remain unchanged

Volumetric reductions of prefrontal cortex have been observed in subjects with schizophrenia, which have correlations with the severity of cognitive and negative symptoms of the disease [61, 62]. There are also inconsistent reports of reduced hippocampal volume in schizophrenia [63]. To examine whether *Slc1a1*^+/-^ mice show brain morphological changes including reduced cortical thickness in prefrontal cortex and reduced size of the hippocampus, we performed morphometric analysis of Nissl-stained sagittal brain sections.

#### Prefrontal cortex

**Fig 2:**
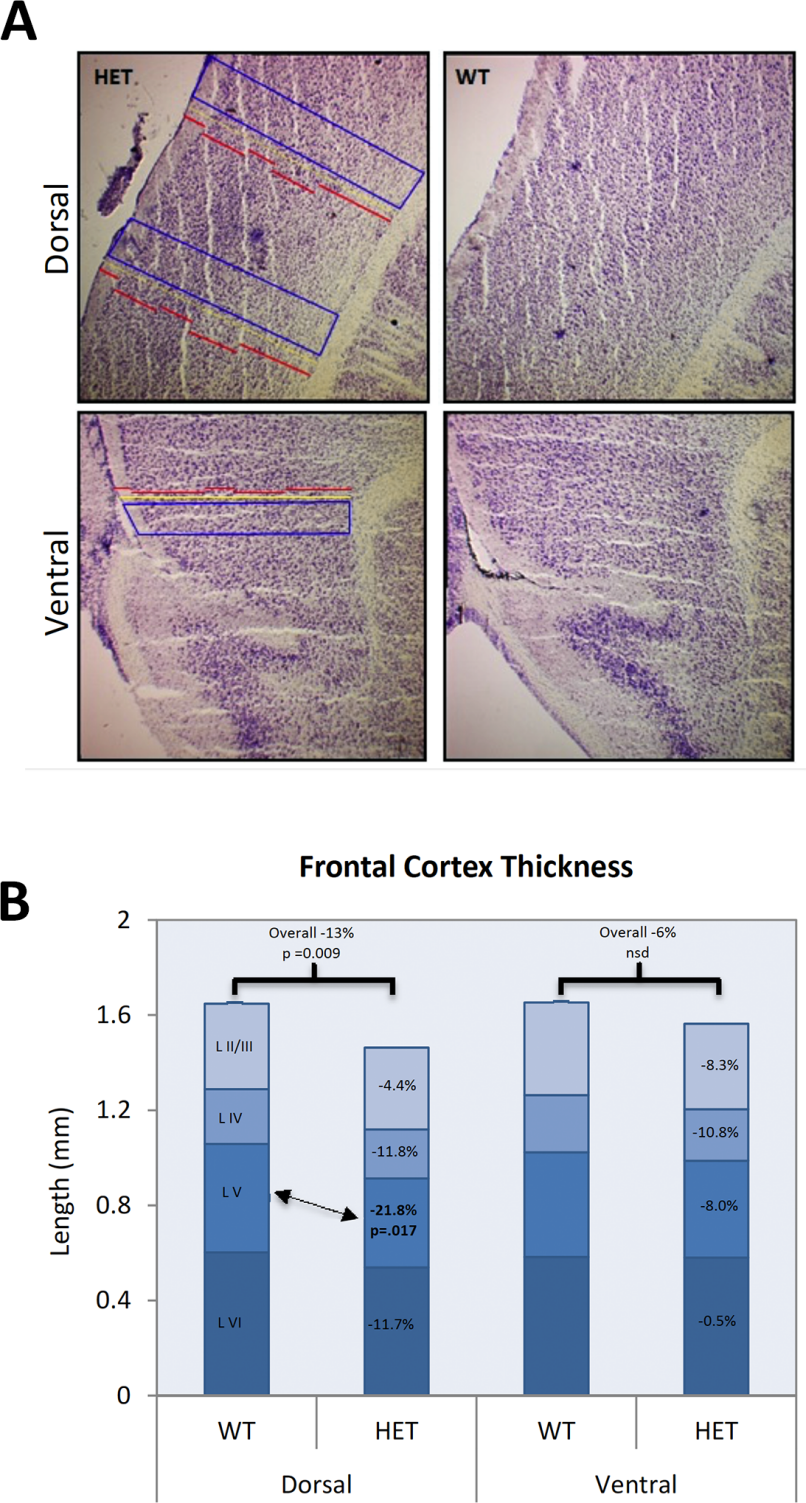
The thickness of DMPFC is reduced in *Slc1a1*^+/-^ mice but VMPFC thickness remains unchanged. Representative images (A) and morphometric analysis (B) of prefrontal cortex in Nissl-stained sagittal brain sections of *Slc1a1*-HET (n = 8) and WT (n = 5) mice. A) The length of individual cortical layers was measured in duplicate on two different sections for each animal, in both the DMPFC and VMPFC areas. B) A two-way (Sex x Genotype) repeated measures ANOVA combining all available measurements for each of the layers as well as the total cortical thickness was performed. *WT*, wildtype; *HET*, *Slc1a1*-heterozygous; *DMPFC*, dorsomedial prefrontal cortex; *VMPFC*, ventromedial prefrontal cortex.

Overall cortical thickness was significantly reduced (F_(1,7)_ = 8.88, p = 0.021) in the DMPFC of *Slc1a1*-HET mice compared to WT mice, but there were no differences in the VMPFC thickness (F_(1,4)_ = 2.76, p = 0.172) between genotypes. We also did not find any significant effects of Sex, Section Level, or Sex x Genotype interactions on overall cortical thickness in either region. The difference in the DMPFC thickness was further supported by a significant post-hoc result (Scheffé p = 0.009). The results of two-way ANOVAs for each cortical layer in the DMPFC and VMPFC indicated that only layer V of the DMPFC was significantly affected due to Genotype (-21.8% reduction; F_(1,8)_ = 7.49, p = 0.0256) **(Fig 2)**. Other layers showed trends for decreased thickness in both regions, but none were significantly affected at the p < 0.05 level.

#### Hippocampus

**Fig 3:**
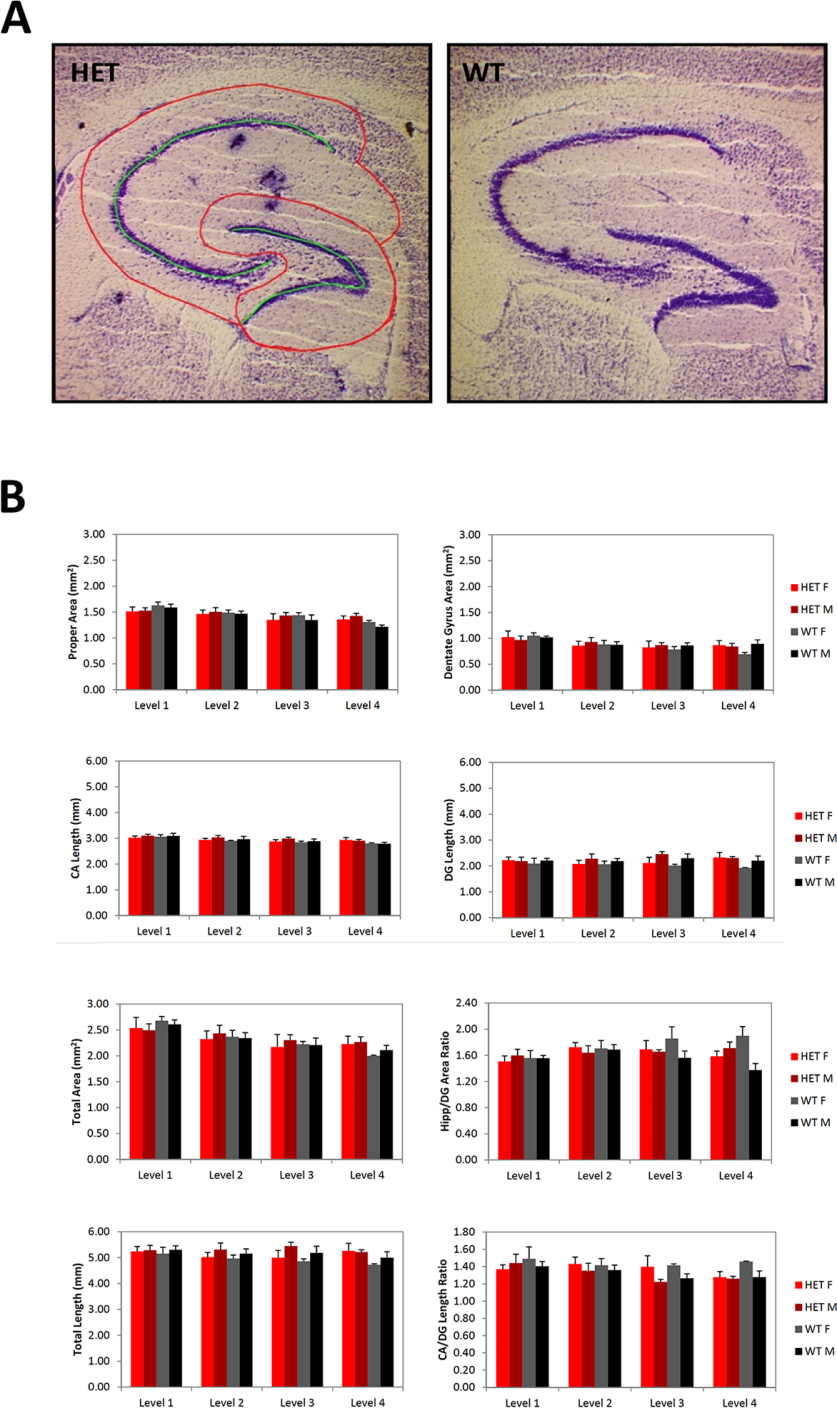
Hippocampal size does not change in *Slc1a1*-HET mice. Representative images (A) and morphometric analysis (B) of hippocampus in Nissl-stained sagittal brain sections of *Slc1a1*-HET (n = 8) and WT (n = 5) mice. A) The total area of the hippocampal formation, the subareas of the hippocampus proper and DG, and the ratio of the hippocampus proper area to the DG area, as well as the length of the stratum pyramidale of the complete CA, the length of the stratum granulosum in the DG, the total length of these combined, and the ratio of CA to DG lengths were measured. These measurements were obtained on 4 section levels through each hippocampus of each animal. B) A two-way (Sex x Genotype) repeated measures ANOVA combining all available measurements for each of the section levels for each animal was performed. *WT*, wildtype; *HET*, *Slc1a1-*heterozygous. *DG*, dentate gyrus; *CA*, cornu ammonis.

Although there was a highly significant effect of Section Level on multiple hippocampal morphometry measures, there were no significant main effects of Genotype or Sex. However, we observed two nominally significant interaction effects. The area of the hippocampus proper exhibited a significant Section Level x Genotype interaction (F_(3,30)_ = 2.99, p = 0.0467) and the ratio of the Hippocampus Proper/ DG areas showed a significant Section Level x Genotype x Sex interaction (F_(3,30)_ = 3.95, p = 0.0173). Inspection of the data revealed that these effects were likely caused by slightly different gradients from medial to lateral regions of the hippocampus in the different animal subgroups **(Fig 3)**. Although the measurements were performed completely blind by a single rater, and the standard errors were acceptably small, we cannot fully exclude the possibility of sampling artifacts contributing to these findings.

### Transcriptional effects

#### RNA expression changes in *Slc1a1+/-* mouse brains are related to synaptic and cognitive functions

To examine the global consequences of *Slc1a1* haploinsufficiency on the expression of other genes, we used a stranded RNA-Seq approach to assess the whole brain and whole blood transcriptomes in *Slc1a1-*HET (n = 5) and WT (n = 5) mice. The average read length was 72 bases with an average quality score of 35. For the brain, an average of 23.3M reads were quantified in each sample, with > 95% alignment to the mm10 mouse genome. Approximately 81% of the aligned reads were classified as exonic, and were located within 23,420 annotated genes. For the whole blood samples, an average of 21.6M reads were obtained per sample, with 83% alignment to the Mm10 genome and 55% classified as exonic. Only genes with a mean count across all samples of 10 or more, and genes whose differential expression result was not determined to be due to a single outlier sample were included in downstream analysis of the brain data (n = 13,542) and blood data (n = 10,160).

Comparisons of gene expression levels between the two genotype groups were made using DE-Seq2 in separate analyses for each tissue type. For the brain, differences in expression were considered significant based on a q value < 0.01 and an absolute difference exceeding +/- 1.25-fold (i.e., log2 change > +/- 0.322). For the blood, differences were considered significant based on a q value < 0.01 and an absolute difference of more than +/- 2-fold (i.e., log2 change > +/- 1.0). For the brain tissue comparisons, a total of 45 genes were found to be differentially expressed in the HET *vs*. WT samples (**Table 2**). These genes included *Slc1a1* itself (q value = 0), which was found to decrease 2-fold (log2 change = -1.0), as well as several other genes with known roles in the brain development, neuronal activity, signaling and transport functions. To further probe the functions of these genes, we performed functional enrichment analysis using the STRING Consortium Database after filtering on an absolute difference exceeding +/- 25% (i.e., log2 change > +/- 0.322). This indicated over-representation of: Biological Process ontologies involved in Cognition as well as Learning or Memory; Cellular Component ontologies involved in Postsynaptic Density, Dendritic Spine, and Neuron Projection; and KEGG Pathway ontologies involved in Glutamatergic Synapse and Amphetamine Addiction (**Table 3**).

**Table 2:** Differentially expressed genes in brain tissue of *Slc1a1*-HET and WT mice.

Gene	Log2 Change	q value	Gene Title
Slc1a1	-1.02	0	Solute carrier family 1 (neuronal/epithelial high affinity glutamate transporter, system Xag), member 1
Scube1	-0.7	2.46E-10	Signal peptide, CUB domain, EGF-like 1
Arc	0.95	1.99E-08	Activity regulated cytoskeletal-associated protein
Dact2	-0.47	0.0001	Dapper homolog 2, antagonist of beta-catenin (xenopus)
Lmo7	-0.55	0.0002	LIM domain only 7
Tbr1	-0.59	0.0003	T-box brain gene 1
Rgs14	-0.53	0.0004	Regulator of G-protein signaling 14
Creld2	0.43	0.0006	Cysteine-rich with EGF-like domains 2
Junb	0.63	0.0009	Jun-B oncogene
G530011O06Rik	-1.07	0.0016	RIKEN cDNA G530011O06 (non-coding RNA gene)
Slc17a7	-0.37	0.0016	Solute carrier family 17 (sodium-dependent inorganic phosphate cotransporter), member 7
Rhpn2	0.46	0.0017	Rhophilin, Rho GTPase binding protein 2
Tnfaip6	0.5	0.0018	Tumor necrosis factor alpha induced protein 6
Dbp	-0.47	0.0028	D site albumin promoter binding protein
Ormdl2	0.43	0.0028	ORM1-like 2 (S. cerevisiae)
Agxt2l1	0.45	0.0028	Alanine-glyoxylate aminotransferase 2-like 1
Ap1m1	-0.66	0.0029	Adaptor-related protein complex AP-1, mu subunit 1
Slitrk5	-0.53	0.003	SLIT and NTRK-like family, member 5
Ccdc28b	0.39	0.0033	Coiled coil domain containing 28B
Slitrk3	-0.32	0.0035	SLIT and NTRK-like family, member 3
Ppp1r3c	0.34	0.0037	Protein phosphatase 1, regulatory (inhibitor) subunit 3C
Cntrob	-0.57	0.0039	Centrobin, centrosomal BRCA2 interacting protein
Zfp948	0.37	0.0042	Zinc finger protein 948
Grin2a	-0.41	0.0057	Glutamate receptor, ionotropic, NMDA2A (epsilon 1)
Slc6a6	-0.53	0.0059	Solute carrier family 6 (neurotransmitter transporter, taurine), member 6
Zfp318	-0.33	0.0059	Zinc finger protein 318
Slit3	-0.49	0.0062	Slit homolog 3 (Drosophila)
Gm14420	0.34	0.0062	Predicted gene 14420
Nr1d1	-0.64	0.0063	Nuclear receptor subfamily 1, group D, member 1
Foxg1	-0.6	0.0063	Forkhead box G1
Ccsap	-0.46	0.0063	RIKEN cDNA 1700054N08 gene
Zfp449	0.33	0.0063	Zinc finger protein 449
P4ha1	0.41	0.0065	Procollagen-proline, 2-oxoglutarate 4-dioxygenase (proline 4-hydroxylase), alpha 1 polypeptide
Efnb2	-0.49	0.0073	Ephrin B2
Sorcs3	-0.44	0.0076	Sortilin-related VPS10 domain containing receptor 3
Lama4	-0.37	0.0076	Laminin, alpha 4
Adipor2	0.42	0.0076	Adiponectin receptor 2
Srsf5	-0.43	0.008	Serine/arginine-rich splicing factor 5
Kalrn	-0.37	0.008	Kalirin, RhoGEF kinase
Lingo1	-0.35	0.008	Leucine rich repeat and Ig domain containing 1
Tcerg1	-0.33	0.008	Transcription elongation regulator 1 (CA150)
Chrm1	-0.47	0.0084	Cholinergic receptor, muscarinic 1, CNS
Prkca	-0.4	0.0085	Protein kinase C, alpha
Tmem86a	-0.41	0.0087	Transmembrane protein 86A
Chpf2	-0.45	0.0089	Chondroitin polymerizing factor 2

**Table 3:** Functional enrichment of 45 differentially expressed genes (from Table 2) in brain tissue of *Slc1a1*-HET and WT mice.

**GO- Biological Process Pathways**				
**Pathway ID**	**Pathway Description**	**Gene Count**	**FDR**	**Genes**
GO:0050890	Cognition	7	0.00399	Arc, Chrm1, Grin2a, Rgs14, Slc17a7, Sorcs3, Tbr1
GO:0007611	Learning or memory	6	0.0187	Arc, Grin2a, Rgs14, Slc17a7, Sorcs3, Tbr1
GO:0007612	Learning	5	0.021	Arc, Grin2a, Rgs14, Sorcs3, Tbr1
GO:0044708	Single-organism behavior	7	0.0325	Arc, Grin2a, Rgs14, Slc17a7, Slitrk5, Sorcs3, Tbr1
**GO- Cellular Component Pathways**				
**Pathway ID**	**Pathway Description**	**Gene Count**	**FDR**	**Genes**
GO:0014069	Postsynaptic density	5	0.0216	Arc, Chrm1, Grin2a, Rgs14, Sorcs3
GO:0043197	Dendritic spine	4	0.0216	Arc, Grin2a, Nr1d1, Rgs14
GO:0030425	Dendrite	6	0.0364	Arc, Chrm1, Grin2a, Nr1d1, Prkca, Rgs14
GO:0043005	Neuron projection	8	0.0364	Arc, Ccsap, Chrm1, Grin2a, Nr1d1, Prkca, Rgs14, Slc17a7
GO:0097458	Neuron part	9	0.0364	Arc, Ccsap, Chrm1, Grin2a, Nr1d1, Prkca, Rgs14, Slc17a7, Sorcs3
**KEGG Pathways**				
**Pathway ID**	**Pathway Description**	**Gene Count**	**FDR**	**Genes**
4724	Glutamatergic synapse	4	0.0212	Grin2a, Prkca, Slc17a7, Slc1a1
5031	Amphetamine addiction	3	0.0362	Arc, Grin2a, Prkca

#### RNA expression changes in *Slc1a1+/-* mouse blood are related to immune activity and inflammation

For the whole blood comparisons, a total of 74 genes were found to be differentially expressed. Interestingly, the most significant of these genes included Jun B (q = 2.7e-22), Jun (q = 1.9e-15), and Fos (q = 4.8e-8) (**Table 4**), which are highly related immediate early genes involved in numerous cellular responses (e.g., stress, inflammation, immune activity). The STRING analysis further probed the functions of all of the differentially expressed blood genes, and indicated over-representation of several Biological Process ontologies involved in immune activation, cytokine responses, inflammation, and drug responses, among others (**Table 5**). Similarly, KEGG Pathway ontologies over-represented in the significantly changed genes included several involved in cytokine signaling, infection, and inflammation (**Table 5**).

**Table 4:** Differentially expressed genes in blood tissue of *Slc1a1*-HET and WT mice.

Gene	Log2 Change	q Value	Gene Title
Junb	1.43	2.70E-22	Jun-B oncogene
Jun	1.51	1.90E-15	Jun oncogene
Sowaha	1.17	2.70E-08	Sosondowah ankyrin repeat domain family member A
Fos	1.46	4.80E-08	FBJ osteosarcoma oncogene
Apol11a	1.68	9.10E-08	Apolipoprotein L 11a
Cirbp	-1.03	1.40E-07	Cold inducible RNA binding protein
1810053B23Rik	1.95	1.40E-07	RIKEN cDNA 1810053B23 gene
Rgs1	1.6	1.90E-07	Regulator of G-protein signaling 1
Lars2	1.42	1.70E-06	Leucyl-tRNA synthetase, mitochondrial
Ccl3	1.58	1.70E-06	Chemokine (C-C motif) ligand 3
Osm	1.47	1.90E-06	Oncostatin M
Gfap	1.15	2.80E-06	Glial fibrillary acidic protein
Ccl4	1.45	9.20E-06	Chemokine (C-C motif) ligand 4
Plk3	1.3	2.70E-05	Polo-like kinase 3
Tbx21	1.18	3.30E-05	T-box 21
Slc16a1	1.04	0.0001	Solute carrier family 16 (monocarboxylic acid transporters), member 1
Fads1	1.04	0.0001	Fatty acid desaturase 1
Dtl	1.17	0.0002	Denticleless homolog (Drosophila)
Cenpi	1.13	0.0002	Centromere protein I
Gm10785	1.15	0.0002	Predicted gene 10785 (Mus musculus)
Sgol1	1.01	0.0003	Shugoshin-like 1 (S. pombe)
Ermap	1.02	0.0003	Erythroblast membrane-associated protein
Tjp1	1.16	0.0003	Tight junction protein 1
Gm166	1.11	0.0003	Predicted gene 166
Vangl1	1.08	0.0004	Vang-like 1 (van gogh, Drosophila)
Tnfrsf9	1.2	0.0004	Tumor necrosis factor receptor superfamily, member 9
Ppp1r15a	1.01	0.0004	Protein phosphatase 1, regulatory (inhibitor) subunit 15A
Irf7	1.01	0.0004	Interferon regulatory factor 7
Wipi1	1.14	0.0005	WD repeat domain, phosphoinositide interacting 1
Rhd	1.07	0.0005	Rh blood group, D antigen
Syt14	1.05	0.0006	Synaptotagmin XIV
Sh3d19	1.07	0.0006	SH3 domain protein D19
Dusp1	1.1	0.0006	Dual specificity phosphatase 1
Snord110	-1.25	0.0006	Small nucleolar RNA, C/D box 110
Cdc6	1.01	0.0006	Cell division cycle 6
Ppap2a	1.12	0.0006	Phosphatidic acid phosphatase type 2A
Slit1	1.2	0.0006	Slit homolog 1 (Drosophila)
Redrum	1.2	0.0007	Redrum, erythroid developmental long intergenic non-protein coding transcript
Mpc2	1.06	0.0007	Mitochondrial pyruvate carrier 2
Fbxo30	1.02	0.0008	F-box protein 30
Darc	1.12	0.0009	Duffy blood group, chemokine receptor
Crat	1.03	0.001	Carnitine acetyltransferase
Kit	1.08	0.0011	Kit oncogene
Scn4a	-1.24	0.0011	Sodium channel, voltage-gated, type IV, alpha
Oas1e	1.31	0.0013	2'-5' oligoadenylate synthetase 1E
Tmod1	1.05	0.0013	Tropomodulin 1
Fcer2a	-1.05	0.0014	Fc receptor, IgE, low affinity II, alpha polypeptide
Prnp	1.08	0.0016	Prion protein
5830428M24Rik	-1.03	0.0017	RIKEN cDNA 5830428M24 gene
Rrm2	1.01	0.0017	Ribonucleotide reductase M2
Ctsf	1.02	0.0018	Cathepsin F
Clnk	1.08	0.0018	Cytokine-dependent hematopoietic cell linker
Tnk1	1.1	0.002	Tyrosine kinase, non-receptor, 1
Mylpf	1.07	0.0021	Myosin light chain, phosphorylatable, fast skeletal muscle
Klri2	1.05	0.0021	Killer cell lectin-like receptor family I member 2
Hist1h2bp	1.12	0.0024	Histone cluster 1, H2bp
Erdr1	-1.13	0.0026	Erythroid differentiation regulator 1
Gypa	1.02	0.0026	Glycophorin A
Cyp4b1-ps2	1.07	0.0029	Cytochrome P450, family 4, subfamily b, polypeptide 1, pseudogene 2
Ifng	1.23	0.003	Interferon gamma
Fasl	1.03	0.0038	Fas ligand (TNF superfamily, member 6)
BC020402	-1.02	0.0043	cDNA sequence BC020402
Crisp2	1.03	0.0044	Cysteine-rich secretory protein 2
Socs2	1.07	0.0047	Suppressor of cytokine signaling 2
Jpx	-1.06	0.005	Jpx transcript, Xist activator (non-protein coding)
Ska1	1.07	0.0051	Spindle and kinetochore associated complex subunit 1
Cib3	1.01	0.0064	Calcium and integrin binding family member 3
Aldh3b2	1.07	0.0065	Aldehyde dehydrogenase 3 family, member B2
Trim7	-1.03	0.0079	Tripartite motif-containing 7
Nphp3	-1.05	0.009	Nephronophthisis 3 (adolescent)

**Table 5:** Functional enrichment of 74 differentially expressed genes (from Table 4) in blood tissue of *Slc1a1*-HET and WT mice.

**GO- Biological Process Pathways**				
**Pathway ID**	**Pathway Description**	**Gene Count**	**FDR**	**Genes**
GO.0043922	Negative regulation by host of viral transcription	3	0.043	Ccl3, Ccl4, Jun
GO.0002376	Immune system process	14	0.047	Ccl3, Ccl4, Clnk, Fasl, Ifng, Irf7, Jun, Junb, Kit, Mylpf, Osm, Rhd, Tbx21, Tnk1
GO.0002763	Positive regulation of myeloid leukocyte differentiation	4	0.047	Ccl3, Fos, Ifng, Jun
GO.0006955	Immune response	10	0.047	Ccl3, Ccl4, Clnk, Fasl, Ifng, Irf7, Kit, Mylpf, Osm, Tnk1
GO.0050900	Leukocyte migration	5	0.047	Ccl3, Ccl4, Ifng, Kit, Tbx21
GO.0002891	Positive regulation of immunoglobulin mediated immune response	3	0.048	Fcer2a, Ifng, Tbx21
GO.0006925	Inflammatory cell apoptotic process	2	0.048	Fasl, Ifng
GO.0009314	Response to radiation	7	0.048	Cirbp, Dtl, Dusp1, Fos, Jun, Junb, Plk3
GO.0030595	Leukocyte chemotaxis	4	0.048	Ccl3, Ccl4, Ifng, Kit
GO.0032496	Response to lipopolysaccharide	6	0.048	Ccl3, Fasl, Fos, Ifng, Jun, Junb
GO.0034097	Response to cytokine	8	0.048	Darc, Fos, Ifng, Jun, Junb, Kit, Osm, Socs2
GO.0042493	Response to drug	7	0.048	Ccl3, Ccl4, Fos, Ifng, Jun, Junb, Prnp
GO.0045672	Positive regulation of osteoclast differentiation	3	0.048	Ccl3, Fos, Ifng
GO.0051591	Response to cAMP	4	0.048	Dusp1, Fos, Jun, Junb
GO.0070887	Cellular response to chemical stimulus	14	0.048	Ccl3, Darc, Dusp1, Ifng, Jun, Junb, Kit, Osm, Plk3, Ppp1r15a, Prnp, Slc16a1, Socs2, Tbx21
GO.1990441	Negative regulation of transcription from RNA polymerase II promoter in response to ER stress	2	0.048	Jun, Ppp1r15a
**KEGG Pathways**				
**Pathway ID**	**Pathway Description**	**Gene Count**	**FDR**	**Genes**
5132	Salmonella infection	6	2.00E-05	Ccl3, Ccl4, Fos, Ifng, Jun, Tjp1
4060	Cytokine-cytokine receptor interaction	7	0.001	Ccl3, Ccl4, Fasl, Ifng, Kit, Osm, Tnfrsf9
4620	Toll-like receptor signaling pathway	5	0.001	Ccl3, Ccl4, Fos, Irf7, Jun
5142	Chagas disease (American trypanosomiasis)	5	0.001	Ccl3, Fasl, Fos, Ifng, Jun
5323	Rheumatoid arthritis	4	0.004	Ccl3, Fos, Ifng, Jun
5168	Herpes simplex infection	5	0.009	Fasl, Fos, Ifng, Irf7, Jun
5144	Malaria	3	0.01	Darc, Gypa, Ifng
4380	Osteoclast differentiation	4	0.012	Fos, Ifng, Jun, Junb
5321	Inflammatory bowel disease (IBD)	3	0.017	Ifng, Jun, Tbx21
5161	Hepatitis B	4	0.018	Fasl, Fos, Irf7, Jun
5140	Leishmaniasis	3	0.019	Fos, Ifng, Jun
5164	Influenza A	4	0.028	Fasl, Ifng, Irf7, Jun

#### Upstream regulator analysis reveals possible common transcriptional regulators of differentially expressed genes in brain and blood

**Fig 4:**
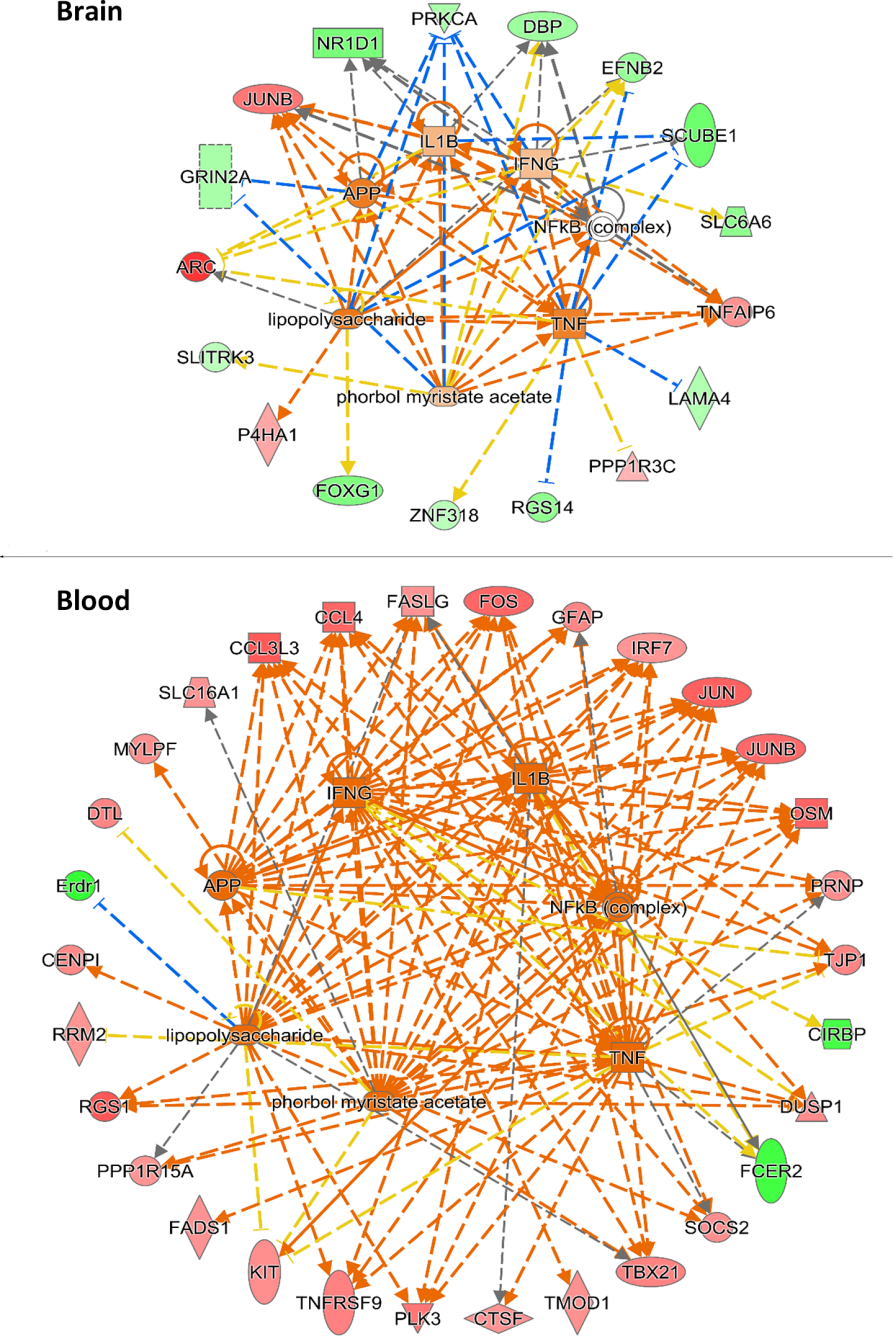
Predicted effects of upstream regulators on the levels of differentially expressed genes in brain and blood of *Slc1a1*^+/-^ mice. IPA software was used to indicate the direction of change and relatedness of the common upstream regulators (n = 7) of the differentially expressed genes in the brain and blood of *Slc1a1*-HET mice. Genes that were changed in expression are shown in the perimeter of each diagram. Genes with significantly increased expression are colored in red and genes with significantly decreased expression are colored in green. The predicted upstream regulators are shown in the center. Orange dashed lines indicate predicted positive effects that are consistent with the observed changes in expression. Blue dashed lines indicate predicted negative effects that are consistent with the observed changes. Yellow dashed lines denote inconsistent predicted and observed effects.

Although the most significantly affected genes differed for brain and blood, we tested whether common upstream regulators might explain part of the overall set of changes that were seen in the two tissue types. Each tissue showed evidence of possible upstream regulators of the most robustly changed genes (**Table 6**). For blood, most of the top predicted upstream regulators were related to immune and proinflammatory pathways. Unexpectedly, for brain, the top predicted upstream regulators included growth factors, dopamine and several related to cellular activity, but also included several related to inflammation and immune activation that were also seen for the blood (**Table 6**, bold entries). The predicted effects of these common upstream regulators were visualized to indicate the direction of change and relatedness to each other (**Fig 4**). This analysis indicated the potential for changes in four common endogenous upstream regulators (APP, IFNG, IL1B, and TNF) to produce several of the observed effects in both tissues, as well as the potential for two exogenous compounds, including lipopolysaccharide (LPS) and phorbol myristate acetate (PMA) to produce similar effects.

**Table 6:** Upstream regulator findings for differentially expressed genes in brain and blood.

**Brain**				
**Upstream Regulator**	**Molecule Type**	**Activation Z-Score**	**P-Value of Overlap**	**Target Molecules in Dataset**
TGFB1	Growth factor	1.94	3.24E-02	GRIN2A, JUNB, P4HA1, PPP1R3C, PRKCA, SLIT3, TNFAIP6
Forskolin	Chemical toxicant	1.92	3.52E-02	ARC, JUNB, PPP1R3C, TNFAIP6
**Lipopolysaccharide (LPS)**	Chemical drug	1.68	1.14E-02	ARC, EFNB2, FOXG1, JUNB, P4HA1, PRKCA, SCUBE1, TNFAIP6
**APP**	Other	1.52	2.23E-02	ARC, GRIN2A, JUNB, NR1D1, PRKCA
Morphine	Chemical drug	1.51	1.24E-04	ARC, GRIN2A, JUNB, SLC1A1
**Tumor Necrosis Factor (TNF)**	Cytokine	1.4	8.44E-04	ARC, EFNB2, JUNB, LAMA4, PPP1R3C, PRKCA, RGS14, SCUBE1, TNFAIP6, ZNF318
Cocaine	Chemical drug	1.22	3.13E-04	ARC, EFNB2, JUNB, Zfp948
CREB1	Transcription regulator	1.07	1.90E-02	ARC, JUNB, SORCS3, Zfp948
LY294002	Kinase inhibitor	1	1.42E-02	ADIPOR2, EFNB2, JUNB, SLC1A1
FGF2	Growth factor	0.96	4.42E-03	ARC, EFNB2, GRIN2A, JUNB
**IL1B**	Cytokine	0.65	5.16E-03	ARC, DBP, JUNB, NR1D1, SCUBE1, TNFAIP6
**Phorbol Myristate Acetate (PMA)**	Chemical drug	0.61	3.41E-03	DBP, EFNB2, GRIN2A, JUNB, PRKCA, SLITRK3, TNFAIP6
**IFNG**	Cytokine	0.58	5.32E-04	ARC, DBP, EFNB2, JUNB, NR1D1, PRKCA, SCUBE1, SLC6A6, TNFAIP6
Ca2+	Chemical—endogenous	0.28	1.39E-03	ARC, DBP, JUNB, NR1D1
L-dopa	Chemical—endogenous	0.15	3.79E-02	ARC, DBP, JUNB, RGS14
**NFkB (complex)**	Complex	0	2.63E-02	DBP, JUNB, NR1D1, TNFAIP6
Beta-estradiol	Chemical—endogenous	-0.21	1.40E-03	ARC, CHRM1, EFNB2, FOXG1, GRIN2A, JUNB, LMO7, PPP1R3C, Srsf5, TNFAIP6
PKC(s)	Group	-0.53	6.42E-04	DBP, JUNB, NR1D1, PRKCA
ESR1	Ligand-dependent nuclear receptor	-1.63	9.79E-03	AP1M1, EFNB2, JUNB, NR1D1, PPP1R3C, SLC6A6, TNFAIP6
2-amino-5-phospho-novaleric acid	Chemical	-1.96	1.51E-04	ARC, DACT2, JUNB, SLITRK3
**Blood**				** **
**Upstream Regulator**	**Molecule Type**	**Activation Z-Score**	**P-Value of Overlap**	**Target Molecules in Dataset**
**Lipopolysaccharide (LPS)**	Chemical drug	3.67	1.31E-09	CCL3L3, CCL4, CENPI, DUSP1, Erdr1, FASLG, FOS, GFAP, IFNG, IRF7, JUN, JUNB, KIT, OSM, PLK3, PPP1R15A, RGS1, SOCS2, TBX21, TNFRSF9
**Tumor Necrosis Factor (TNF)**	Cytokine	3.54	2.77E-13	CCL3L3, CCL4, CTSF, DUSP1, FADS1, FASLG, FCER2, FOS, GFAP, IFNG, IRF7, JUN, JUNB, KIT, OSM, PLK3, PPP1R15A, PRNP, RGS1, RRM2, SOCS2, TBX21, TJP1, TNFRSF9
**Phorbol Myristate Acetate (PMA)**	Chemical drug	3.38	4.50E-12	CCL3L3, CCL4, DTL, DUSP1, FASLG, FOS, IFNG, IRF7, JUN, JUNB, KIT, MYLPF, OSM, PPP1R15A, PRNP, RGS1, SLC16A1, TBX21, TJP1
Poly rI:rC-RNA	Biologic drug	3.35	4.93E-10	CCL3L3, CCL4, DUSP1, FASLG, FOS, IFNG, IRF7, JUN, JUNB, Oas1d, PPP1R15A, RGS1
**IL1B**	Cytokine	3.31	1.78E-10	CCL4, CTSF, DUSP1, FASLG, FCER2, FOS, IFNG, IRF7, JUN, JUNB, OSM, PLK3, SOCS2, TBX21, TJP1, TNFRSF9
IL2	Cytokine	3.24	8.18E-10	CCL3L3, CCL4, CDC6, FASLG, FOS, GFAP, IFNG, JUN, KIT, OSM, SOCS2, TBX21, TNFRSF9
**IFNG**	Cytokine	3.2	9.52E-10	CCL3L3, CCL4, CIRBP, DUSP1, FASLG, FCER2, FOS, GFAP, IFNG, IRF7, JUN, JUNB, OSM, PRNP, SOCS2, TBX21, TJP1, TMOD1
**NFkB (complex)**	Cytokine	3.2	2.11E-09	CCL3L3, CCL4, FASLG, FCER2, FOS, GFAP, IFNG, IRF7, JUN, JUNB, KIT, PLK3, TNFRSF9
CSF2	Cytokine	2.95	9.21E-10	CCL3L3, CCL4, FOS, IFNG, OSM, PPP1R15A, PRNP, RRM2, SGO1, SKA1, SOCS2, TNFRSF9
CD3	Complex	-2.87	3.54E-06	CCL3L3,CCL4,FASLG,FOS,IFNG,JUN,JUNB,SOCS2,TBX21,TNFRSF9
IKBKB	Kinase	2.8	7.31E-10	CCL3L3, CCL4, CDC6, CTSF, FASLG, FOS, IFNG, JUNB, SOCS2, TNFRSF9
RELA	Transcription regulator	2.78	3.77E-07	DUSP1, FASLG, FOS, IFNG, IRF7, JUN, JUNB, KIT, PLK3
P38 MAPK	Group	2.76	1.00E-09	CCL3L3, DUSP1, FASLG, FCER2, FOS, IFNG, IRF7, JUN, JUNB, MYLPF, TBX21
**APP**	Other	2.76	1.09E-06	CCL3L3, CCL4, FASLG, FOS, GFAP, IFNG, IRF7, JUN, JUNB, OSM, PRNP, TJP1
TCR	Complex	2.75	3.06E-09	CCL3L3, CCL4, FASLG, FOS, IFNG, IRF7, JUN, JUNB, SOCS2, TBX21
IL1	Group	2.71	1.69E-06	CCL4, DUSP1, FASLG, FOS, GFAP, IFNG, JUN, KIT
CD40	Transmembrane receptor	2.7	2.38E-09	CCL4, DUSP1, FASLG, FCER2, FOS, IFNG, JUN, TBX21, TNFRSF9
IL21	Cytokine	2.62	2.37E-07	CCL3L3, CCL4, FASLG, IFNG, IRF7, SOCS2, TBX21
MAP2K1	Kinase	2.62	2.47E-07	CCL3L3, CCL4, DUSP1, FASLG, FOS, JUN, OSM

Entries in bold are common between brain and blood.

### Biochemical changes

#### Glutathione redox imbalance in the brain of *Slc1a1*^+/-^ mice

**Fig 5:**
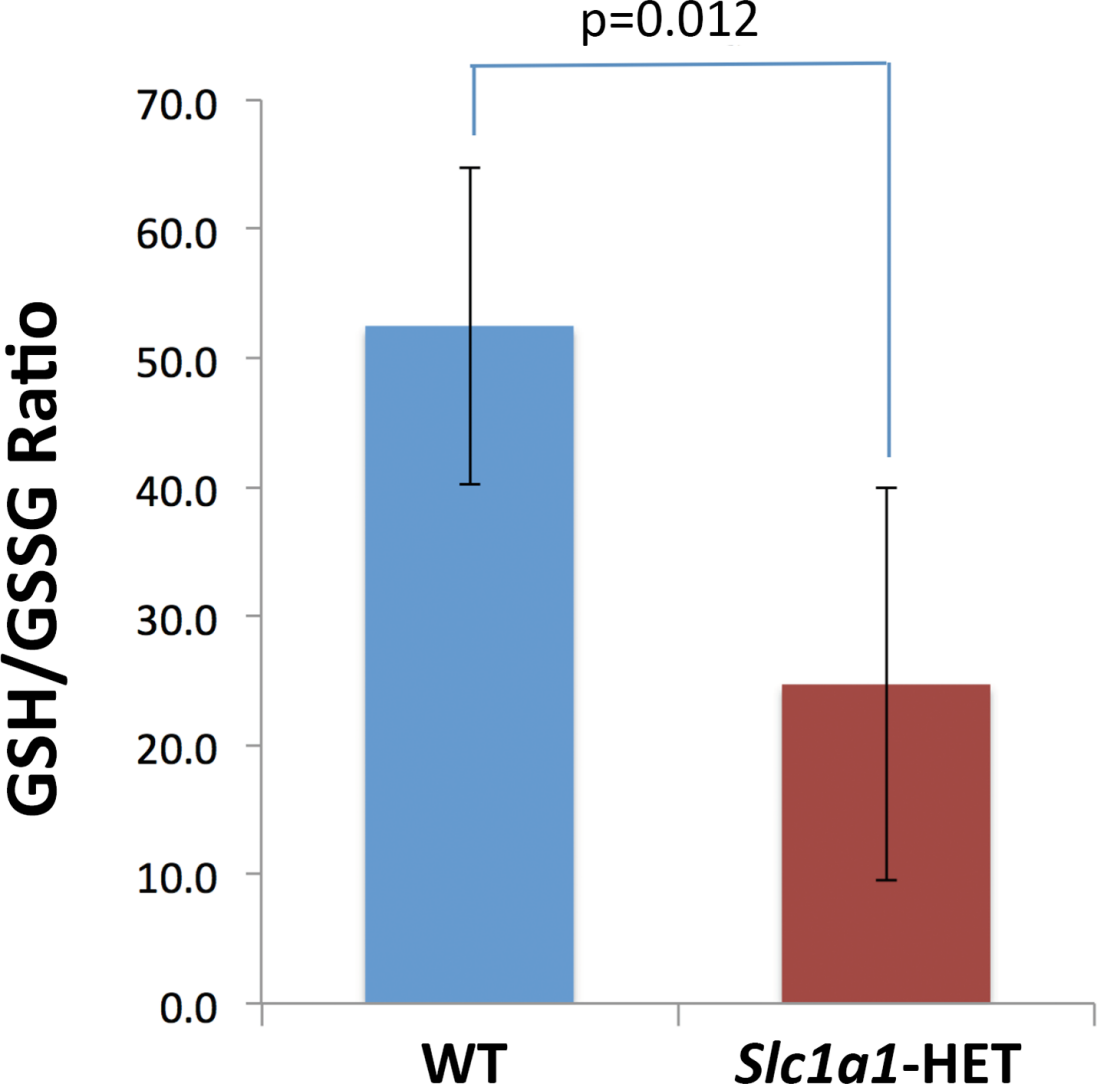
Decreased ratio of GSH/GSSG in *Slc1a1-*HET mice. The ratio of reduced to oxidized glutathione concentration (GSH/GSSG) in the brain tissue of *Slc1a1*-HET mice (n = 5) was found to be almost half of that in WT mice (n = 5). Data are shown as mean ± SEM. *WT*, wildtype; *HET*, heterozygous; *GSH*, reduced glutathione; *GSSG*, glutathione disulfide.

A decreased ratio of reduced to oxidized glutathione concentration (GSH/GSSG) is an indicator of cellular toxicity that can occur under oxidatively stressful conditions [64]. Moreover, this type of glutathione redox imbalance has been consistently reported in subjects with psychiatric disorders [65]. Consistent with these findings, we found that the ratio of GSH/GSSG in the brains of *Slc1a1*-HET mice is almost half of what detected in WT mice (p = 0.012) **(Fig 5)**, implicating a potential redox imbalance and higher vulnerability to oxidative stress in response to *Slc1a1* haploinsufficiency.

#### Increased levels of oxidative DNA damage marker in *Slc1a1*^+/-^ mice

**Fig 6:**
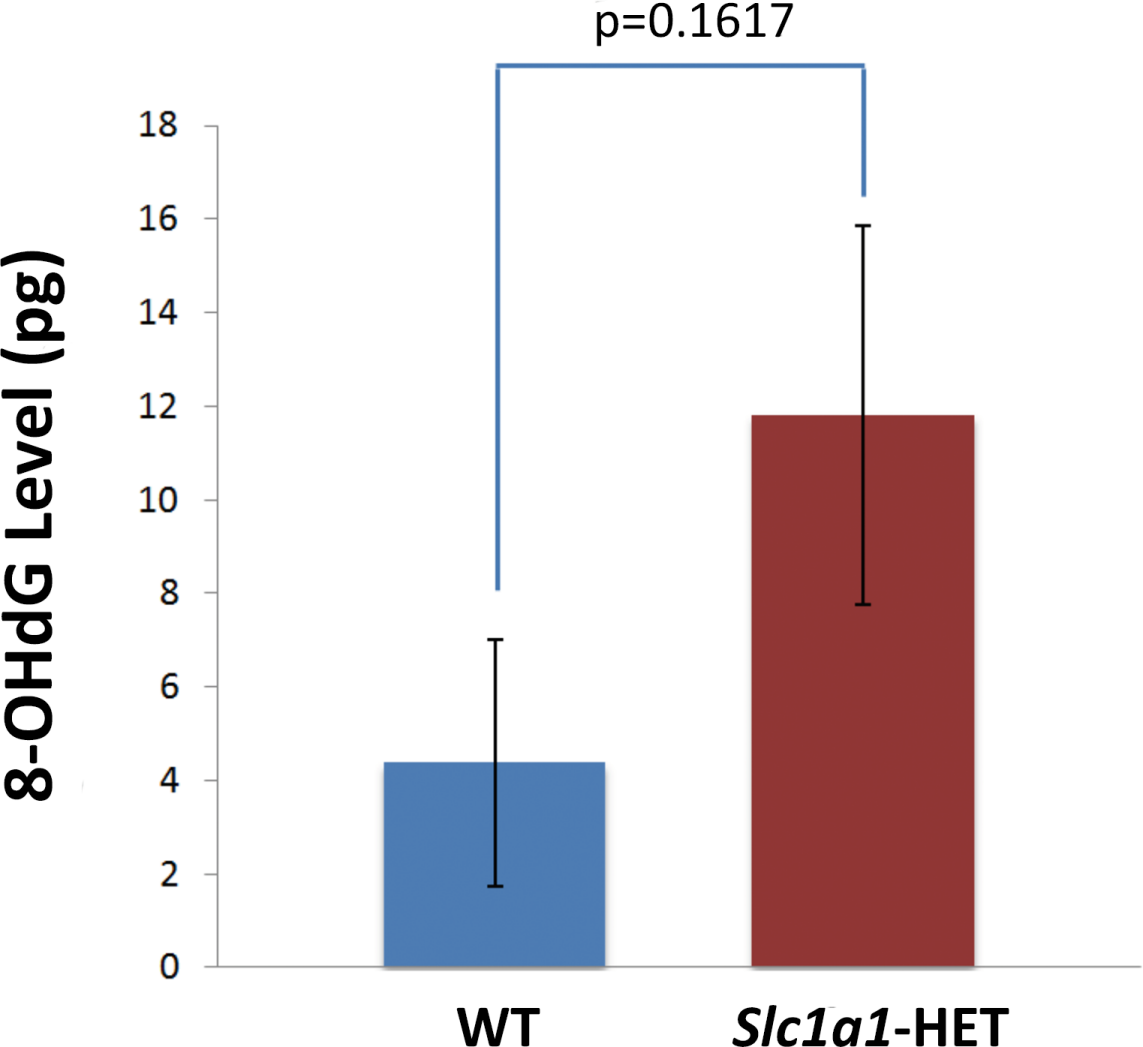
A trend for higher levels of 8-OHdG in *Slc1a1-*HET mice. The level of oxidative DNA damage marker, 8-OHdG, in the brain tissue of *Slc1a1*-HET mice (n = 5) and WT mice (n = 5) was not statistically significant. Data are shown as mean ± SEM. *WT*, wildtype; *HET*, heterozygous; 8-hydroxy-2′-deoxyguanosine.

Based on the glutathione results, we next sought to examine whether *Slc1a1* deficiency might cause oxidative DNA damage in the brain. The data from the 8-OHdG quantification kit indicated a trend for 2.7-fold higher concentration of this oxidative DNA damage marker in the brains of *Slc1a1*-HET mice compared to WT mice **(Fig 6)**. However, the difference was not significant. Although the results are merely suggestive, they might indicate increased vulnerability of *Slc1a1-*HET mouse brain cells for DNA damage as a consequence of impaired cellular oxidant metabolism.

#### Slc1a1 haploinsufficiency does not alter brain apoptosis

Although the exact role of apoptosis in schizophrenia remains uncertain, there have been reports of dysregulation of apoptosis and possible increase in apoptotic susceptibility in several brain regions of individuals with schizophrenia [66]. On the contrary, accumulating evidence suggests that apoptotic activity may be downregulated in chronic schizophrenia [67, 68], although this effect may be influenced by administration of anti-psychotic medications (which can have anti-inflammatory effects). On the other hand, EAAT3 has been suggested to have direct anti-apoptotic activity in neurons [69]. Thus, to address possible changes in apoptosis in Slc1a1-HET and WT mice, we measured the levels of active caspase-3 and phospho-BAD in the brains of a subset of the same mice used in behavioral phenotyping and morphometric experiments. Our data did not indicate any significant differences in the levels of caspase-3 or phospho-BAD between the two genotype groups (Slc1a1-HET and WT). However, we detected a non-significant trend for increased levels of active caspase-3 in male Slc1a1^+/-^ mice, compared to male WT mice (data not shown). The analysis also did not reveal any significant effects of Sex or Sex x Genotype interaction on the levels of either apoptosis marker. Since the calculated concentrations of active caspase-3 and phospho-BAD were normalized to the levels of beta-tubulin, the findings cannot be attributed to different amounts of total protein in the lysates.

#### Increased production of cytokines in *Slc1a1*^+/-^ mice

**Fig 7:**
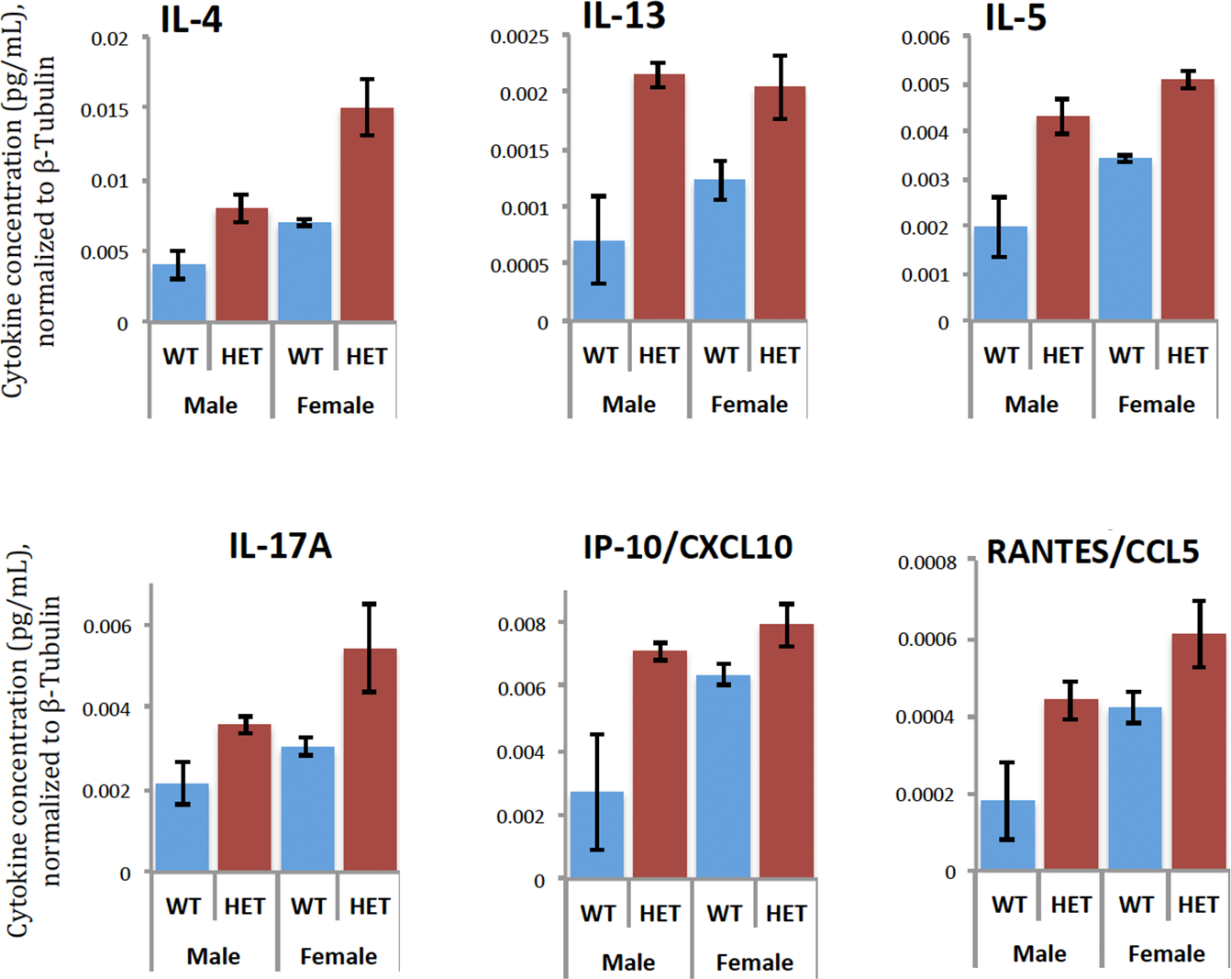
Cytokines with different concentrations in *Slc1a1*-HET *vs*. WT mouse brains. Cytokine profiling assay was performed in the brains of a subset of the same *Slc1a1*-HET and WT mice used for behavioral phenotyping and morphometry (n = 4 males and 4 females per genotype, n = 16 total). To control for different amounts of total protein in the lysates, we normalized all of the cytokine concentrations to the levels of beta-tubulin in each sample. Comparisons between the groups were performed using a 2-way ANOVA (Sex x Genotype) with FDR correction of Genotype main effect p values and a Scheffé post-hoc test used to control for possible non-normality of the ratio data. Only those cytokines surviving FDR correction (q<0.25) are shown. *WT*, wildtype; *HET*, *Slc1a1*-heterozygous.

Cytokines are one of the most important components of the immune system that mediate the response to infectious and other exogenous insults [70]. There is strong evidence for abnormal expression levels of some cytokines in both the brain and peripheral blood of patients with schizophrenia, which have culminated in a cytokine-based model of the disease [41, 71]. Based on the behavioral and brain morphological changes we observed in the *Slc1a1*-HET mice, as well as the observations of over-represented immune pathways in the RNA sequencing data and altered brain redox status, we next sought to determine whether there was evidence of neuroinflammation in the brains of *Slc1a1-*HET mice by measurement of cytokine protein levels. Our results indicated a significantly increased production of proinflammatory cytokines, IL-4, IL-5, IL-13, 1L-17A, IP-10/CXCL10, and RANTES/CCL5 in the brains of *Slc1a1*-HET mice, compared to WT mice **(Table 7; Fig 7)**. Since the calculated cytokine concentrations in each sample were normalized to the levels of β-tubulin, the observed differences cannot be attributed to different amounts of total protein in the lysates. We also found a significant effect of Sex on the concentrations of a subset of the above cytokines (IL-4, IL-5, IP-10 and RANTES), although it did not follow a consistent pattern among different cytokines **(Table 7)**. Interestingly, we also found significant correlations between many of the cytokine levels in the samples **(Table 8)**.

**Table 7:** Results of cytokine profiling assay in *Slc1a1*-HET *vs*. WT mouse brains.

**IL-4**								
	**Mean Square**	**F-Value**	**P-Value**	**Power**	**Scheffé's post hoc test**			
**Sex**	6.88E-05	9.341	0.0109	0.804		**Mean Diff.**	**Crit. Diff.**	**P-Value**
**Genotype**	1.26E-04	17.129	0.0016	0.973	**F, M**	0.00405	0.00309	0.0149
Sex * Genotype	1.66E-05	2.247	0.162	0.266	**HET, WT**	0.00569	0.00309	0.0019
**IL-13**								
	**Mean Square**	**F-Value**	**P-Value**	**Power**	**Scheffé post hoc test**			
**Sex**	1.62E-07	0.73708	0.4089	0.11917		**Mean Diff.**	**Crit. Diff.**	**P-Value**
**Genotype**	4.72E-06	21.53244	0.0007	0.99249	**F, M**	0.00011	0.00053	0.6724
Sex * Genotype	3.88E-07	1.76939	0.2104	0.21916	**HET, WT**	0.00109	0.00053	0.0009
**IL-5**								
	**Mean Square**	**F-Value**	**P-Value**	**Power**	**Scheffé post hoc test**			
**Sex**	4.43E-06	10.99198	0.0069	0.86694		**Mean Diff.**	**Crit. Diff.**	**P-Value**
**Genotype**	0.00001	36.50142	< .0001	0.99994	**F, M**	0.00093	0.00072	0.0165
Sex * Genotype	4.28E-07	1.06073	0.3252	0.15001	**HET, WT**	0.00189	0.00072	0.0001
**IL-17A**								
	**Mean Square**	**F-Value**	**P-Value**	**Power**	**Scheffé post hoc test**			
**Sex**	1.00E-05	4.78509	0.0512	0.50654		**Mean Diff.**	**Crit. Diff.**	**P-Value**
**Genotype**	0.00001	9.44245	0.0106	0.80873	**F, M**	0.00126	0.00137	0.0667
Sex * Genotype	8.58E-07	0.59603	0.4564	0.10589	**HET, WT**	0.00186	0.00137	0.0123
**IP-10/ CXCL10**								
	**Mean Square**	**F-Value**	**P-Value**	**Power**	**Scheffé post hoc test**			
**Sex**	2.00E-05	7.11	0.0219	0.68314		**Mean Diff.**	**Crit. Diff.**	**P-Value**
**Genotype**	0.00003	12.74488	0.0044	0.91371	**F, M**	0.00191	0.00183	0.0417
Sex * Genotype	0.00001	2.80018	0.1224	0.32112	**HET, WT**	0.00272	0.00183	0.0074
**RANTES/ CCL5**								
	**Mean Square**	**F-Value**	**P-Value**	**Power**	**Scheffé post hoc test**			
**Sex**	1.55E-07	7.60029	0.0187	0.71373		**Mean Diff.**	**Crit. Diff.**	**P-Value**
**Genotype**	1.81E-07	8.87796	0.0125	0.78282	**F, M**	0.00019	0.00016	0.0281
Sex * Genotype	3.78E-09	0.18528	0.6752	0.06753	**HET, WT**	0.0002	0.00016	0.0183

Two way (Sex x Genotype) ANOVA, followed by Scheffé post hoc test. Only cytokines with significantly different concentrations that also survived FDR correction of the significant main effect of Genotype at q < 0.25 are displayed. Note that Scheffé post hoc tests confirmed the differences between Het and WT groups for all 6 of these cytokines.

**Table 8:** Correlation of differentially expressed cytokines in *Slc1a1*-HET and WT mice brains.

	IL-4	IL-13	IL-5	IL-17A	IP-10/ CXCL10	RANTES/ CCL5
**IL-4**		0.0014	< .0001	< .0001	0.001	< .0001
**IL-13**	**0.710**		< .0001	< .0001	< .0001	0.0003
**IL-5**	**0.794**	**0.799**		0.0006	< .0001	< .0001
**IL-17A**	**0.906**	**0.796**	**0.739**		< .0001	< .0001
**IP-10/CXCL10**	**0.721**	**0.816**	**0.844**	**0.793**		< .0001
**RANTES/ CCL5**	**0.831**	**0.765**	**0.805**	**0.907**	**0.938**	

Pearson correlation coefficients (lower left half) and their statistical significance (top right half) between different cytokines in *Slc1a1*-HET and WT mouse brains. Bold coefficient values were statistically significant (p < 0.05).

#### *SLC1A1* knockdown or overexpression affects neural cytokine release following oxidative or inflammatory challenge

**Fig 8:**
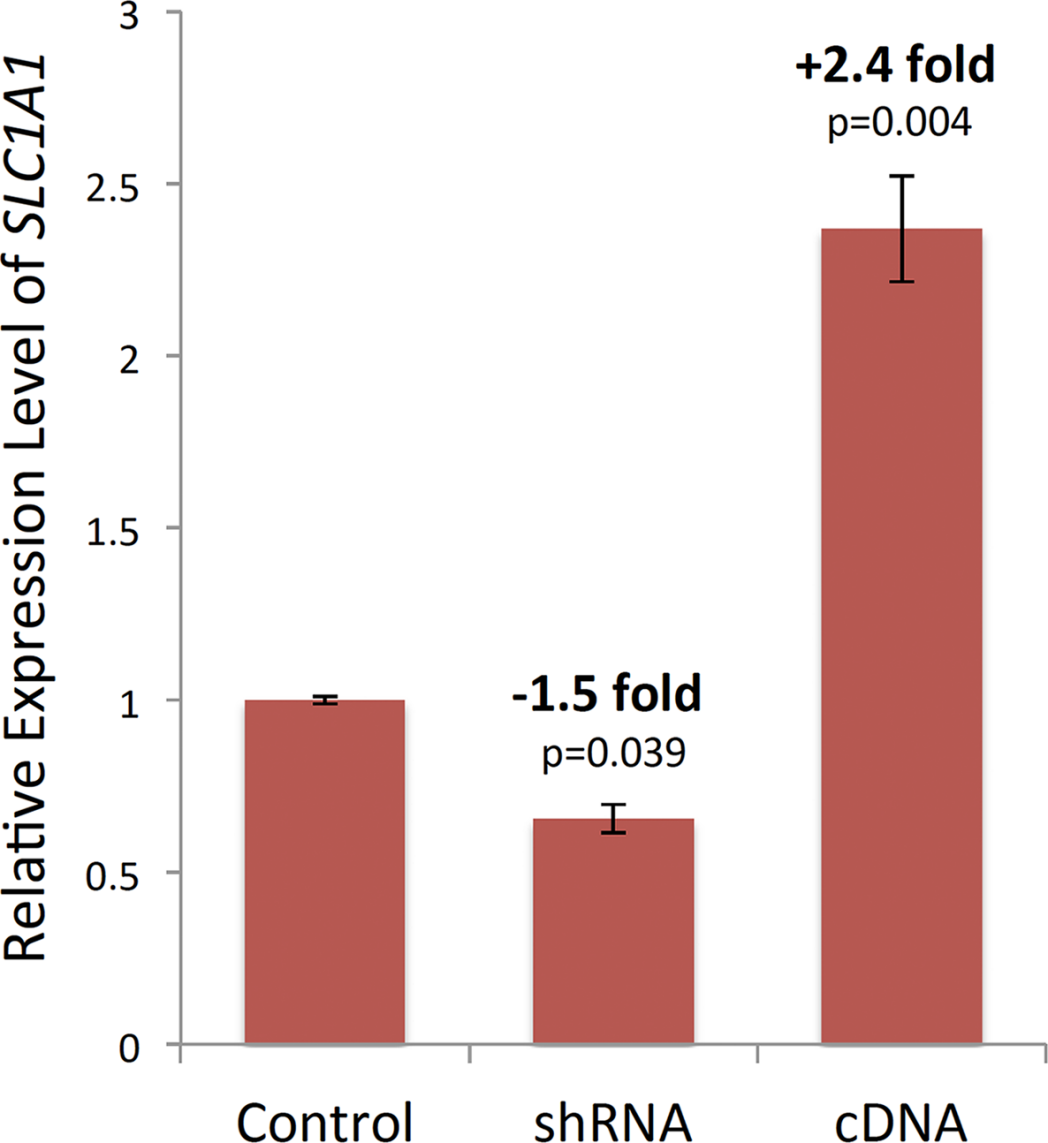
Confirmation of *SLC1A1* expression changes in transfected differentiated SK-N-SH cells. Using real-time RT-qPCR, we confirmed that in SK-N-SH cells treated with *SLC1A1* shRNA or cDNA-containing plasmids, the expression level of this gene significantly decreased or increased, respectively. When normalized to the *SLC1A1* expression level in the non-target shRNA treated SK-N-SH cells (Control), the cocktail of three validated human MISSION *SLC1A1* shRNAs had knocked down the *SLC1A1* mRNA expression by 53%. Conversely, *SLC1A1* cDNA had increased the mRNA expression level of this gene by 2.4-fold.

We next examined how overexpression or knockdown of *SLC1A1* might affect the response of differentiated human neuroblastoma SK-N-SH cells to oxidative stress or pro-inflammatory stimuli using rotenone or LPS treatment. The choice of LPS was particularly appropriate given the evidence from the upstream regulator analysis of brain and blood in *Slc1a1*-HET mice that LPS could produce some of the same gene expression changes that were observed. Using real-time PCR, we first confirmed that in SK-N-SH cells treated with *SLC1A1* shRNA or cDNA-containing plasmids, the expression level of this gene was significantly decreased (-1.53 fold, p = 0.039; **Fig 8**) or increased (+2.4 fold, p = 0.004; **Fig 8**), respectively.

**Fig 9:**
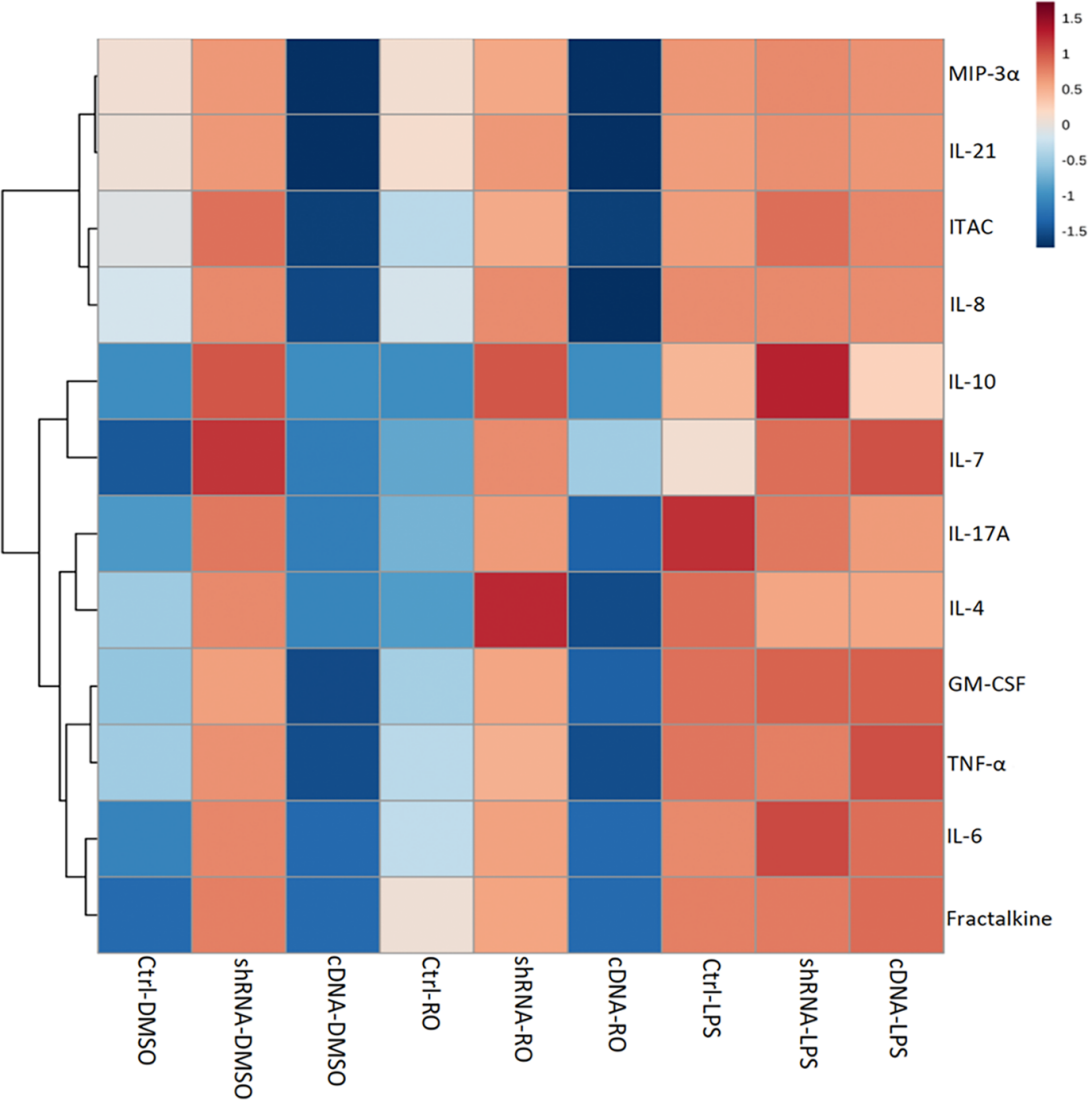
*SLC1A1* knockdown or overexpression affects the neural cytokine release following oxidative or inflammatory challenge. The differentiated SK-N-SH cells subjected to overexpression or knockdown of *SLC1A1* were treated with either LPS (100ng/mL) or rotenone (100nM) or 0.01% DMSO as the vehicle. Using a cytokine profiling assay, the levels of different cytokines in the supernatants of each transfection/ treatment condition were measured. The cytokine results are displayed after hierarchical clustering. Two-way ANOVA indicated a significant effect of *SLC1A1* expression level (shRNA or cDNA plasmid constructs) and treatment (LPS, RO or vehicle) on all but one cytokine (IL-10) and a significant effect of their interaction on several of the cytokines studied (GM-CSF, TNFalpha, MIP-3alpha, IL-4, IL-6, IL-21). *Ctrl*, control (non-target shRNA transfection); *RO*, rotenone; *LPS*, lipopolysaccharide.

Our results from cytokine profiling assay indicated that when SK-N-SH cells were exposed to the complex I inhibitor rotenone or the vehicle (DMSO), *SLC1A1* expression level displayed an inverse relationship with released cytokine/chemokine levels. In other words, *SLC1A1* knockdown increased and *SLC1A1* overexpression decreased the release of several inflammatory cytokines or chemokines under baseline conditions or in response to rotenone, including Fractalkine, ITAC, TNFalpha, GM-CSF, MIP-3alpha, and interleukins 4, 6, 7, 8, 10, 17A and 21 **(Fig 9)**. However, the expression level of *SLC1A1* did not appear to have any consistent effects on cytokine production following the stimulation with LPS, suggesting that the anti-inflammatory functions of *SLC1A1*/EAAT3 might not be sufficient to overcome the effects of the potent inflammatory agent LPS. A two-way ANOVA (Construct x Treatment) of the data confirmed a significant effect of *SLC1A1* construct (overexpression or knockdown) and cell culture treatment (LPS, rotenone or vehicle) on all of the above cytokines, except IL-10, as well as a significant effect of their interaction on a subset of the cytokines studied (GM-CSF, MIP-3alpha, IL-4, IL-6, and IL-21; **Table 9**).

**Table 9:** Main and interaction effects of Construct and Treatment on the cytokine levels in differentiated SK-N-SH cells.

Cytokine	Construct	Treatment	Interaction
**GM-CSF**	6.45e-07	6.11e-13	0.0018
**TNF-alpha**	2.03e-03	3.85e-09	8.49e-05
**MIP-3alpha**	4.95e-06	4.39e-09	0.0488
**IL-6**	8.28e-06	2.65e-07	0.0376
**IL-21**	7.28e-06	1.69e-06	0.0326
**ITAC**	8.28e-06	3.79e-05	0.0551
**Fractalkine**	0.0374	5.86e-05	0.0786
**IL-17A**	0.0189	0.0007	0.1653
**IL-8**	0.0059	0.0028	0.1799
**IL-4**	0.0016	0.0029	0.0326
**IL-7**	0.0002	0.0039	0.0731
**IL-10**	0.0391	0.5897	0.9764

## Discussion

In this report, we describe the first behavioral, anatomical, biochemical and transcriptional phenotypic analysis of late adolescent/young adult *Slc1a1*^+/-^ mice. Our results lend considerable support to the potential utility of a mouse *Slc1a1* haploinsufficiency model for mimicking the *SLC1A1* hemi-deletion CNV we previously described in an extended Palauan family that co-segregated with schizophrenia and psychosis [43]. Our results are also highly consistent with the cellular phenotypic analysis we previously completed on the CNV [44].

The present study has the following major findings: 1) *Slc1a1*^+/-^ mice appear to display several behavioral features that are consistent with well-established endophenotypes of schizophrenia and psychosis including anxiety-like behavior, impaired working memory, decreased exploratory activity and impaired sensorimotor gating. Moreover, these same effects appear to be present in KO *Slc1a1*^-/-^ mice and are an equivalent magnitude; 2) *Slc1a1*^+/-^ mice display reduced thickness of DMPFC, particularly layer V, which is also consistent with neuroanatomical findings in schizophrenia; 3) *Slc1a1*^+/-^ mice exhibit a lower ratio of reduced to oxidized glutathione (GSH/GSSG) as well as a possible trend for increased oxidative DNA damage in the brain; 4) There appears to be compensatory or downstream effects in the brains of *Slc1a1*^+/-^ mice, that lead to significant changes in the expression of genes and pathways involved in synaptic and cognitive functions; 5) There are robust changes in the expression of RNAs related to immune activation and inflammation in the blood of *Slc1a1*^+/-^ mice; 6) The DMPFC of *Slc1a1*^+/-^mice shows evidence of increased production of inflammatory cytokines; and 7) *SLC1A1* knockdown in human neuroblastoma cells increases, while *SLC1A1* overexpression decreases, the release of several cytokines in response to an oxidative stress-inducing reagent. We now discuss some of the implications of these findings.

### Behavioral and brain morphological changes of *Slc1a1*^+/-^ mice

Most rodent models of schizophrenia have behavioral phenotype changes that resemble the positive-like symptoms of the disease, likely reflecting altered mesolimbic dopamine function. However, some models also show learning and memory impairment, analogous to cognitive symptoms of schizophrenia [72]. Using well-established behavioral paradigms that model aspects of schizophrenia [52], we found several phenotypes consistent with those seen in this disease in *Slc1a1*^+/-^ mice.

#### Increased anxiety-like behavior

In the EPM test, *Slc1a1*^+/-^ and *Slc1a1*^-/-^ mice showed a non-significant tendency to enter closed arms more frequently and spend significantly longer time in those arms than WT mice. Although these differences were not significant, in the OF test, *Slc1a1*^+/-^ and *Slc1a1*^-/-^ mice also spent significantly less time in the anxiety-provoking center zone of the chamber compared to WT mice. The EPM test is one of the most popular tests for animal models of anxiety [52, 73]. It is believed that the aversion of mice to explore the open arms of the maze is caused by fear of open and elevated spaces, indicative of anxiety-like phenotype [74]. Similarly, the OF test assesses novel environment exploration and general locomotor activity, and provides a screen for anxiety-related behavior in mice. The results suggest that *Slc1a1* deficiency might promote anxiety-like behavior in mice.

It is well known that anxiety disorders contribute to the etiology of psychiatric disorders, including schizophrenia [75]. Interestingly, *SLC1A1* was the first positional candidate gene in the anxiety disorder obsessive compulsive disorder (OCD) [76, 77]. Among OCD positional candidate genes, *SLC1A1* has among the strongest support for its association with this disorder [78–84]. Additionally, variations in *SLC1A1* gene have also been associated with other forms of anxiety disorders, including posttraumatic stress disorder (PTSD) or anxiety symptom severity in autism spectrum disorder (ASD) [85, 86]. Thus, our observation of anxiety-like phenotype of *Slc1a1*^+/-^ mice further supports the association of this gene with anxiety and its potential comorbidity with schizophrenia.

#### Impaired working memory and reduced thickness of DMPFC

Of the cognitive impairments in schizophrenia, substantial research has focused on working memory, typically defined as the ability, in the absence of sensory cues, to maintain and process information over short periods of time [87, 88]. The prefrontal cortex has been suggested as the primary site of working memory and subjects with schizophrenia, who are considered to have prefrontal cortical dysfunction, have demonstrated deficits on a variety of working memory tests [54]. Many parts of the brain, including hippocampus and basal forebrain, are thought to assist the prefrontal cortex in the performance of working memory tasks, particularly in rodents [89].

In our study, the *Slc1a1*^+/-^ and *Slc1a1*^-/-^ mice showed significantly impaired spontaneous alternation in the Y-maze test compared to WT mice. This paradigm is based on rodents’ innate tendency to explore novel environments and alternate between maze arms without any reinforcement and thereby, provides a measure of spatial working memory. Our gene expression data on the brains of *Slc1a1*^*+/-*^ mice provides one possible explanation for these behavioral observations, based on the evidence for differential expression of genes related to synaptic function, cognitive function, and learning and memory in these mice.

In humans, performance on working memory tasks depends, at least in part, upon the sustained firing of pyramidal cells in layers III and V of dorsolateral prefrontal cortex (DLPFC) [90]. There is evidence for aberrant activation of this brain region during working memory tasks in schizophrenic patients [88, 91]. Volumetric reductions of prefrontal cortex have also been reported in subjects with schizophrenia, which are correlated with the severity of cognitive and negative symptoms [61, 62]. Human postmortem studies have suggested that layers III and V of the DLPFC, which give rise to glutamatergic projections to neostriatum and pontine nuclei, demonstrate structural and transcriptome alterations in schizophrenia, including reports of a significant decrease in the thickness of cortical layer V in individuals with schizophrenia [92–95]. Interestingly, these layers overlap the localization of EAAT3-positive neurons in the forebrain, which are more numerous in projection layers III and V [7].

In support of this evidence, we found a significant 15% reduction in DMPFC overall thickness in *Slc1a1*^+/-^ mice, but no change in overall brain mass or brain:body weight ratios. Although we detected the reduction in all cortical layers of the DMPFC of *Slc1a1*^+/-^ mice, it was most prominent in cortical layer V, the layer that contains large numbers of glutamatergic projection neurons. Many researchers have debated the homology between specific regions in primate and rodent forebrain. For example, the classically-defined DLPFC region of the primate brain does not exist in the rodent brain. However, it is believed that the rodent medial prefrontal cortex shares homology with the DLPFC region of the human brain [96, 97].

Overall, our results in the working memory task as well as in the morphometric and gene expression analyses support the association of *Slc1a1* deficiency with cognitive impairments observed in schizophrenia. Our findings also lend support to the existing evidence in translational neuroscience regarding the cognitive tasks as behavioral outcome measures in animal models of psychosis and the potential involvement of rodents’ PFC circuitry in cognitive deficits seen in animal models of human diseases.

#### Decreased exploratory activity

In rodents, the NOR task, as measured by the amount of time taken to explore a new object in the presence of the familiar object, has become a benchmark task for assessing recognition memory. The hippocampus is involved in memory processing, recognition and storage of the contextual details and temporal order of previous experiences [98], and thus has been proposed as the main structure involved in objection recognition memory in rodents [99]. We did not observe any gross morphological changes in cellular lamina or areal measures in the hippocampus of *Slc1a1*^+/-^ mice, and we did not find any significant differences in performance of the NOR task in these mice. However, both *Slc1a1*^+/-^ and *Slc1a1*^-/-^ mice showed significantly diminished overall object exploratory activity as measured by total time spent in proximity to the objects. Although replicating negative symptoms of schizophrenia, such as anhedonia and avolition in animal models is difficult, reduced exploratory behavior is suggested as a measure of anhedonia in mice. In other words, it has been suggested that anhedonic mice display decreased duration of object exploration compared to control mice in the new object exploration paradigm [100]. Thus, we can speculate that decreased exploration of the objects in *Slc1a1*^+/-^ mice might indicate their reduced interest in the objects, implying an anhedonic state in these animals. Alternatively, it is possible that the reduced interaction with the objects was related to reduced attention or to increased anxiety in *Slc1a1*^+/-^ mice, which also spent less time in the center of the open field chamber.

#### Impaired sensory gating function

As the final behavioral phenotype examined, we found that in *Slc1a1*^+/-^ and *Slc1a1*^-/-^ mice, PPI of the acoustic startle reflex decreased to approximately half of that in WT mice. PPI is believed to reflect the sensorimotor gating process that occurs in the first few hundred milliseconds prior to conscious attention, filtering out weak and unimportant stimuli and preventing sensory overload during a sensory task. Therefore, PPI is believed to reflect pre-attentive processing [58, 72]. Reduced PPI is commonly accepted as an endophenotype of schizophrenia, which models the positive symptoms of the disease across various species [59, 57, 101]. Our finding of a highly significant reduction in PPI (~48%) in *Slc1a1*^+/-^ mice, suggests the importance of *Slc1a1*/EAAT3 in sensorimotor gating functions of the brain. Given our previous finding of the association of *Slc1a1* disruptions with schizophrenia, this observation further supports the idea that PPI is likely to represent the “interface of psychosis and cognition" [57].

### Biochemical imbalances in the brain of *Slc1a1*^+/-^ mice

#### Altered glutathione redox state

EAAT3 is distinct from other EAATs in that besides transporting glutamate, it is also responsible for neuronal uptake of cysteine, the rate-limiting substrate for the synthesis of intracellular glutathione (GSH) [9, 102, 103]. GSH is the principal redox buffer in the CNS, protecting it against oxidative stress-induced cell damage and its deficiency is associated with neural cell death and neurodegenerative diseases [15, 104]. The significance of oxidative stress in the pathophysiology of schizophrenia is now well established [23, 105]. In fact, it has been proposed that different etiological factors of schizophrenia impose their effect by inducing cellular oxidative stress and damage [106]. Moreover, glutathione redox imbalances have been consistently reported in subjects with psychiatric disorders [65].

Given that a decreased ratio of reduced to oxidized glutathione concentration (GSH/GSSG) is an indicator of cellular toxicity and oxidative stress [64, 107], our finding of a significantly lower ratio of GSH/GSSG in *Slc1a1*^+/-^ mice implicates a potential redox imbalance and higher vulnerability to oxidative stress in the brain as a result of *Slc1a1* haploinsufficiency. Our results complement the previous reports of a decreased neuronal GSH content, increased oxidant levels and neurodegeneration in *Slc1a1*-null mice [11, 17, 19, 20] and indicates that even a partial loss of EAAT3 can make the cells vulnerable to oxidative stress. In addition, the expression of EAAT3 is known to increase following oxidative stress through the Nrf2-antioxidant responsive element (ARE) pathway [108]. When considered alongside these reports, our findings further implicate overexpression of EAAT3 as a *bona fide* neuroprotective mechanism to resist oxidative stress.

#### Increased oxidative DNA damage

One of the proposed mechanisms by which oxidative stress is involved in the pathology of schizophrenia is through oxidative DNA damage as a result of excessive ROS [109, 110]. The oxidative DNA damage might, in turn, stimulate cell cycle arrest and ultimately, cause cell death via apoptosis or necrosis [53]. In support of this idea, there is evidence for increased levels of 8-OHdG, a robust marker of oxidative DNA damage, in post-mortem hippocampus of patients with poor-prognosis schizophrenia [111]. In agreement with these reports, we found a trend for increased oxidative DNA damage in the brains of *Slc1a1*^+/-^ mice.

#### No significant changes in the level of apoptosis markers

In contrast to the significant changes in redox status and possible elevation in DNA damage, we did not detect any changes in the level of apoptosis, as determined by active caspase-3 and phospho-BAD measurements in the brains of *Slc1a1*^+/-^ mice. Such findings are consistent with our additional observation of no change in overall brain mass or brain:body weight ratios in *Slc1a1*^+/-^ mice.^-^The exact role of apoptosis in schizophrenia and the direction of its changes in the disease remain controversial [68]. On one hand, there have been reports of dysregulation of apoptosis and possible increase in apoptotic susceptibility in several brain regions of individuals with schizophrenia. On the other hand, accumulating evidence suggests that apoptotic activity may be downregulated in chronic schizophrenia [66–68]. In support of the latter hypothesis, the levels of active caspase-3, which is the most important effector caspase involved in neuronal apoptosis, have been reported to be normal or even slightly decreased in the cortex of subjects with schizophrenia [112]. This observation is in contrast with what seen in neurodegenerative disorders, such as Alzheimer's and Parkinson's diseases [68]. It is possible that downregulation of apoptotic activity in chronic schizophrenia could be influenced by antipsychotic medications, which have anti-inflammatory effects as well. In support of this hypothesis, studies have suggested that atypical antipsychotic pretreatment attenuated the rate of apoptosis in neural cells *in vitro* [113] and antipsychotic treatment in schizophrenia was associated with higher cortical levels of the anti-apoptotic marker Bcl-2, compared to antipsychotic-naïve subjects [114].

#### Increased expression of inflammation-related genes and production of cytokines

An emerging theory of schizophrenia derives from substantial evidence from neuroanatomical, environmental and genetic studies, which suggest that inflammation and immune dysfunction are involved in the pathogenesis of schizophrenia [41, 70, 71]. Cytokines and chemokines can play both pro- and anti-inflammatory roles and are among the most important components of the immune system, capable of penetrating the blood-brain barrier (BBB) to permit cross-talk between the CNS and immune system. However, when the balance of these inflammatory mediators is disrupted, they can induce neuronal inflammation, damage and degeneration, which can manifest in a variety of neuropsychiatric disorders [41, 71, 115].

In support of this idea, there is evidence that increased levels of inflammatory cytokines, following a genetic insult or exposure to oxidative stress or infectious agents, can contribute to cognitive, negative, and positive symptoms in schizophrenia [40]. Elevated levels of cytokines have been reported in postmortem brain and peripheral blood of subjects with schizophrenia [115, 116, 117]. Neuroimaging studies have shown active inflammation in the brains of patients with psychosis [118]. Conversely, subjects with psychosis exhibit reduced symptoms when administered anti-inflammatory agents [23, 119]. Furthermore, in genome-wide association studies, schizophrenia's strongest association with genetic markers has been observed in the major histocompatibility complex (MHC) locus [120, 121]. The recent report of excessive complement C4 activity in the development of schizophrenia [42] adds to this body of evidence, which has collectively led to the emergence of a cytokine model for schizophrenia.

We found evidence for inflammation and immune dysfunction in *Slc1a1*^+/-^ mice at both transcriptional and protein levels. At the protein level, our results suggested increased production of several inflammatory cytokines in the DMPFC of *Slc1a1*^+/-^ mice. Two of the cytokines we profiled, IL-4 and IL-17A, were observed to change in the same direction in both *Slc1a1*^+/-^ mice and following knockdown of *SLC1A1* in SK-N-SH cells. In contrast, overexpression of *SLC1A1* in SK-N-SH cells decreased the release level of these cytokines. Notably, the levels of these two cytokines in our samples were also highly correlated (R = 0.906). IL-4 is the key cytokine of the type 2 immune response and has been extensively studied in the context of its role in immunity. Accumulating evidence suggests that it plays a critical role in the regulation of cognitive functions of the brain, such as memory and learning [122]. Moreover, it has been suggested that cognitive task performance leads to the accumulation of IL-4–producing T cells in the meninges, and IL-4-null mice exhibit cognitive deficits [123].

Our whole transcriptome profiling studies of the brain and blood lend considerable support to the likelihood that there might be widespread immune activation or heightened inflammation in *Slc1a1*-deficient mice. Our data also point to several key molecules that might serve as master upstream regulators of these specific transcriptional responses in both the blood and brain. Manipulation of at least one of these predicted regulators–LPS–produced effects *in vitro* that were similar in nature to those seen in the mouse brain and blood. Although overexpression of *SLC1A1* appeared highly effective at reducing the proinflammatory state produced by oxidative challenge with rotenone, we cannot claim that it was similarly effective at preventing the striking inflammatory effect caused by LPS. Nonetheless, future research that examines the potential anti-inflammatory effects of SLC1A1 overexpression may prove fruitful under more physiological conditions in human subjects. Taken together, our findings support a potential anti-inflammatory function of *SLC1A1*/EAAT3.

## Limitations and conclusions

Our behavioral and biochemical results are largely based on a small number of mice studied during a single point in time, and should therefore be viewed as preliminary in nature, particularly the findings regarding morphometric outcomes. Nonetheless, they support the critical role of EAAT3 in neuronal glutathione synthesis and maintenance of the brain redox state and further support the role of glutathione and oxidative stress in the pathophysiology of schizophrenia. At least part of this role appears to involve oxidative damage of DNA and creation of a proinflammatory state in the brain and periphery.
